# Tumor initiation and early tumorigenesis: molecular mechanisms and interventional targets

**DOI:** 10.1038/s41392-024-01848-7

**Published:** 2024-06-19

**Authors:** Shaosen Zhang, Xinyi Xiao, Yonglin Yi, Xinyu Wang, Lingxuan Zhu, Yanrong Shen, Dongxin Lin, Chen Wu

**Affiliations:** 1 Department of Etiology and Carcinogenesis, National Cancer Center/National Clinical Research Center/Cancer Hospital, Chinese Academy of Medical Sciences and Peking Union Medical College, 100021 Beijing, China; 2 Key Laboratory of Cancer Genomic Biology, Chinese Academy of Medical Sciences and Peking Union Medical College, 100021 Beijing, China; 3 Changping Laboratory, 100021 Beijing, China; 4 Collaborative Innovation Center for Cancer Personalized Medicine, Nanjing Medical University, Nanjing, 211166 China; 5 Sun Yat-sen University Cancer Center, State Key Laboratory of Oncology in South China, Guangzhou, 510060 China; 6 CAMS Oxford Institute, Chinese Academy of Medical Sciences, 100006 Beijing, China

**Keywords:** Cancer microenvironment, Cancer genetics

## Abstract

Tumorigenesis is a multistep process, with oncogenic mutations in a normal cell conferring clonal advantage as the initial event. However, despite pervasive somatic mutations and clonal expansion in normal tissues, their transformation into cancer remains a rare event, indicating the presence of additional driver events for progression to an irreversible, highly heterogeneous, and invasive lesion. Recently, researchers are emphasizing the mechanisms of environmental tumor risk factors and epigenetic alterations that are profoundly influencing early clonal expansion and malignant evolution, independently of inducing mutations. Additionally, clonal evolution in tumorigenesis reflects a multifaceted interplay between cell-intrinsic identities and various cell-extrinsic factors that exert selective pressures to either restrain uncontrolled proliferation or allow specific clones to progress into tumors. However, the mechanisms by which driver events induce both intrinsic cellular competency and remodel environmental stress to facilitate malignant transformation are not fully understood. In this review, we summarize the genetic, epigenetic, and external driver events, and their effects on the co-evolution of the transformed cells and their ecosystem during tumor initiation and early malignant evolution. A deeper understanding of the earliest molecular events holds promise for translational applications, predicting individuals at high-risk of tumor and developing strategies to intercept malignant transformation.

## Introduction

**Fig. 1:**
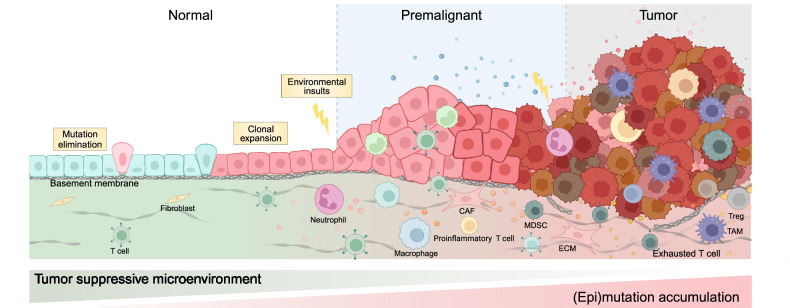
Multistage tumorigenesis. In normal tissue, somatic mutations sporadically arise and either are eliminated by tumor-suppressive mechanisms or gain proliferative advantages to form clones. The mutant clones can still maintain homeostasis until they are exposed to additional stimulus. Their proliferation becomes uncontrolled, and malignant transformation initiates, progressing from premalignant lesions to advanced tumors. During this process, the transformed cells gradually accumulate additional genetic mutations and epigenetic alterations, exhibiting increasingly malignant traits such as immune evasion, structural disruption, and invasion. Simultaneously, the microenvironment of these cells evolves from being tumor-suppressive to supportive of malignancy. This includes dysfunctional immunosurveillance, the emergence of tumor-promotive inflammation, gradual transformation of fibroblasts to CAFs, as well as stiffening of the ECM. CAF cancer associated fibroblast, TAM tumor associated macrophages, MDSC myeloid-derived suppressor cell, ECM extracellular matrix. Created with BioRender.com

It is generally believed that tumorigenesis is a multi-stage process, wherein the initial step is the occurrence of an oncogenic mutation in a single somatic cell. The mutation endows cells with clonal advantages, allowing the mutant clone to expand and accumulate additional genetic and epigenetic alterations, ultimately resulting in an irreversible, highly heterogeneous, and invasive lesion^1^ (Fig. 1). Mutations that confer growth competitiveness and promote cancer evolution are referred to as cancer driver mutations. Identifying driver mutations and revealing their roles in tumors represent key areas of focus in cancer genome research. Recent advancements in sampling and sequencing technologies facilitate the detection of somatic mutations and clonal expansion in normal tissues. It is surprising that even though driver mutations harbored by positively selected clones overlap to a great extent with cancer driver mutations and are pervasive in morphologically normal tissues, only a low annual incidence rate of cancer is diagnosed in populations. It is suggested that mutations alone are insufficient for tumor formation, and other prerequisite molecular events need to be identified. Additionally, humans have evolved various strategies to maintain homeostasis and defend oncogenic transformation. However, environmental insults and aging often disrupt the balance and increase the risk of cancer formation.^2,3^ Although the mechanisms of these risk factors contributing to cancer progression have been widely explored, how they are involved in early tumorigenesis and interact with specific oncogenic mutations are still not completely understood. The non-genetic effects of external signaling may explain the paradox of genetic mutation and tumorigenesis. Epigenetic rewiring can serve as another impetus to release uncontrollable growth and survival potential.

Cells capable of forming a neoplastic phenotype after acquiring genetic and epigenetic alterations will henceforth be referred to as “transformed cells”. Their clonal evolution is the result of a balance between intrinsic competency and extrinsic selective pressures, which is influenced by neighboring competitors, the microenvironment, and the cooperative tissue architecture. It used to be difficult to detect the rare precursors of tumors, while being armed with innovative technology, the identities of transformed cells and their interactions with the environment are being elucidated. In this review, we explore the driver events that enhance the transforming competency of a cell into full-fledged tumors, and examine the key transitions underlying tumor initiation and early tumorigenesis driven by these events. In addition, given that numerous interventional strategies for advanced tumors are limited by their heterogeneity, premalignant stage is regarded as a promising timing for intervention.^4^ Therefore, we also summarize how the molecular processes can be utilized to predict patients who are at high-risk of developing consequential cancer, and to develop preventive strategies that intercept malignant transformation.

## The research history of tumor initiation and early tumorigenesis

**Fig. 2:**
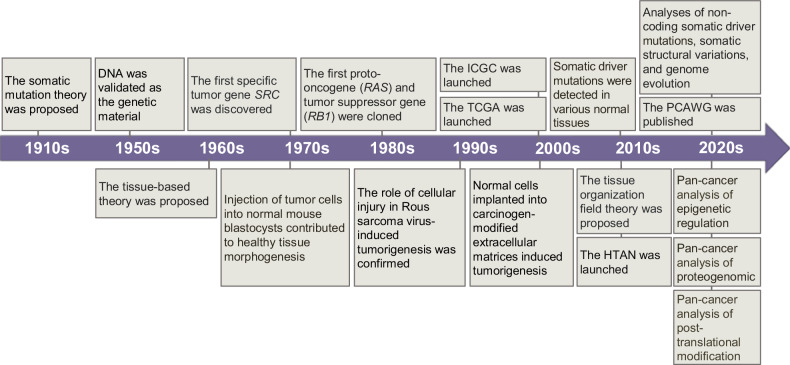
Research history of tumor initiation and early tumorigenesis. The upper section emphasizes the role of somatic mutations in tumorigenesis, while the lower section demonstrates the evidence of the driver events beyond genetic events. ICGC the International Cancer Genome Consortium, TCGA the Cancer Genome Atlas, PCAWG the Pan-Cancer Analysis of Whole Genomes, HTAN the Human Tumor Atlas Network

The earliest explanation for the origin of cancer can be dated back to the early 1900s, cell-free extracts of a diseased animal were able to transmit tumors to healthy animal, suggesting that tumors originate from a unit smaller than a cell^5^ (Fig. 2). In 1914, Theodor Boveri proposed the somatic mutation theory after observing chromosomal abnormalities in tumor cells.^6^ Subsequent studies validated DNA as the genetic material and revealed that tumorigenesis requires the accumulation of approximately six or seven mutations.^7,8^ The term “oncogene” was introduced in 1960s when genetic material of certain viruses was verified to contribute to malignant transformation.^9^ The first specific tumor gene was identified in 1976 by Michael Bishop and Harold Varmus, that part of the DNA of avian sarcoma virus hybridized in the genomes of birds transforming normal cells to tumor cells, and named it as *SRC*.^10^ This indicated that the genetic material in our genome is capable of transforming normal cells. Subsequently, the first proto-oncogene, *RAS*, and tumor suppressor gene, *RB1*, were cloned in the early 1980s.^11–13^ Following this, a significant number of these two classes of cancer genes were identified, accompanied by discovery of other forms of variations, including copy number alterations, translocations and promoter hypermethylation.^14^ In the middle of 2000s, benefiting from next-generation sequencing, cancer genomics flourished and promoted the launch of large-scale tumor sequencing initiatives, such as The Cancer Genome Atlas (TCGA) in 2006 and the International Cancer Genome Consortium (ICGC) in 2007.^15^ The TCGA consortium published its Pan-Cancer Analysis of Whole Genomes (PCAWG) data in 2020, which contained the whole genomic sequencing data of 38 tumor types from more than 2800 patients, largely expanding our understanding of cancer genomics.^16^ According to the influence in cancer development, mutations can be categorized as driver mutations and passenger mutations. The driver mutations confer fitness advantage for clone expansion while other preexisting mutations, lacking positive selection properties, are referred to as passenger mutations,^17^ and over 3,000 cancer driver genes have been identified experimentally or computationally to date.^18^ Notably, in the last decade, deep sequencing from low-input samples has helped to identify somatic mutations in normal tissues, which are highly concordant with the tumor driver mutations.^19^ It reveals a limitation of the somatic mutation theory, that is the mere presence of mutations is insufficient for tumorigenesis, suggesting that there are other driver events.

On the other hand, Victor A. Triolo first proposed that cancer is a tissue-based disease in 1965.^20^ Following studies have verified that the capability of mutated malignant cells to induce tumors is context-dependent. Injecting tumor cells into normal mouse blastocysts can result in the development of normal embryos, indicating that malignant cells alone do not necessarily lead to tumors.^21^ The role of tissue injury in Rous sarcoma virus-mediated tumorigenesis,^22,23^ and tumors induced by carcinogen-treated extracellular matrices^24,25^ both further confirmed that extrinsic factors influence the outcome of tumorigenesis. Accordingly, tissue organization field theory was proposed in 2011.^26^ The theory posits that aberrant tissue organization and cell-cell interactions contribute to tumorigenesis, with carcinogens targeting the entire tissue. In 2018, the Human Tumor Atlas Network (HTAN) was launched,^27^ aiming at setting three dimensional atlases at crucial transitions of multiple tumors, including tumor initiation and local expansion, based on single-cell and spatial methods, and elucidating complex interactions between cells and their dynamic tumor ecosystem. It is expected to help us better understand how microenvironmental factors and transformed cells cooperatively promote the early transformation. Furthermore, the pan-cancer analysis of epigenome, transcriptome, proteome, and post-translational modification were recently published,^28–34^ providing multidimensional information of the tumor biology and possibly giving insights for the research of tumorigenesis.

## Molecular drivers of tumorigenesis

### Genetic alterations

#### Single nucleotide variants

Single nucleotide variants continuously accumulate through lifespan, originating from errors during DNA replication and repair processes, resulting from both endogenous factors (e.g., cellular metabolites, reactive oxygen species, nitrogen species, and transposable elements) and exogenous factors (e.g., radiation, tobacco, alcohol, and other chemical mutagens). Spontaneous chemical modifications can also serve as mutagens.^35,36^ Somatic mutations in morphologically normal tissues can establish a baseline for studying cancer genome evolution and for identifying key drivers of malignant transformation. In recent years, a series of studies have analyzed the mutational landscape across nonmalignant tissues, shedding light on tissue-specific mutational burdens, mutational signatures, and the spectrum and frequency of driver mutations and their clonal expansions (Table 1), which can be influenced by stem cell dynamics, tissue turnover patterns, and environmental exposures.^3,19^ Mutational signatures, developed to depict various DNA damage and repair processes, offer insights into mutagenic mechanisms.^35^ It shows that age-related signatures, such as single base substitution signature 1 (SBS1) and SBS5, are prevalent across phenotypically normal tissues, although their contributions vary.^37,38^ These signatures are the primary mutagenic factors in most types of tissues, especially those with high rates of cellular proliferation, such as the intestines.^37,38^ In contrast, exogenous mutational signatures often play a relatively minor role. However, there are some exceptions, such as the SBS22 mutational signature associated with aristolochic acid, which is common in the liver samples^37^ and is also significantly enriched in the urothelial samples from Chinese donors.^39^

**Table 1:** Dynamics of genome evolution in tumorigenesis

Tissue	Normal	Precancerous	Tumor	Evolution	Refs
Blood	DNMT3A, TET2, TP53, CUX1, ASXL1, SRSF2, EZH2, SF3B1, JAK2, BCORL1	**MDS:** SF3B1, TET2, SRSF2, ASXL1, DNMT3A, RUNX1 **MDS**: ASXL1, DNMT3A, BCOR, SRSF2, U2AF1, TET2 **MF**: TP53	**CMML:** TET2, SRSF2, ASXL1, KRAS, ZRSR2, CBL, RUNX1 **AML:** SFRS2, TET2, DNMT3A, AXSL1, TP53, BCORL, RUNX1, BCORL1	DNMT3A and PPM1D mutations are more frequent in CHIP than in myeloid malignancies; TP53 mutation increases from MF to AML.	^104,135,348,355,457,458^
Bladder	KMT2D, KDM6A, ARID1A, RBM10, EP300, STAG2, NOTCH2, CDKN2A	Not reported	TP53, MLL2, ARID1A, KDM6A, CDKN2A, PIK3CA, YWHAZ, NCOR1	TP53 mutations are prevalent in cancer but are rare in normal urothelium.	^39,459^
Bronchus	TP53, NOTCH1, FAT1, ARID1A, ARID2, CHEK2, PTEN, IDH1, CREBBP, EP300	**ADH:** KRAS, BRAF, ERBB2, PDGFRA, HERC2, PDGFRA, ARID1A	**ADC:** EGFR, RBM10, TP53, KRAS, LRP1B, STK11, BRAF, HERC2	Not reported	^162,460^
Colon	NFKBIZ, ARID1A, PIGR, ERBB3, ERBB2, AXIN2, FBXW7, PIK3CA, STAG2	**UC dysplasia:** TP53, RNF43, BRAF, APC, KRTAP4, CTNNB1, KRAS **SSL:** BRAF, KRTAP4, MK167 **AD:** APC, MK167, APOB, KRAS, LRP1B, FAT2	TP53, KRAS, APC, PIK3CA, SMAD4, FBXW4, SOX9, RNF43, ARID1B	The frequency of TP53 mutations increases during CRC tumorigenesis. APC mutations occur from the precancerous state and are not detected in normal colons	^117,137,334,347^
Esophagus	NOTCH1, TP53, FAT1, NOTCH2, PPM1D, ZFP36L2, NOTCH3, CHEK2	**Squamous dysplasia:** TP53, NOTCH1, ZFHX4, CDKN2A, FAT1, ZNF750 **BE**:TP53, CDKN2A, KDM6A, ARID1A, ARID1B, APC, ERBB2	**ESCC:** TP53, NOTCH1, KDM6A, KMT2D, LRP1B, ZNF750, CDKN2A, SMAD4, CCSER1, SOX2, BCL6, CCDN1 **EAC:** TP53, CDKN2A, ARID1A, EYS, SYNE1, ABCB1	**ESCC:** The Frequency of TP53 mutations increases and NOTCH1 mutations decreases during ESCC tumorigenesis. **EAC:** The frequencies of TP53 and ERBB2 mutations increase from BE to EAC.	^54,59,461–465^
Endometrium	ERBB2, ERBB3, PIK3CA, ARHGAP35, PIK3R1, KRAS, FBXW7, PPP2R1A, ZFHX3, FOXA2	**AEH:** PTEN, ARID1A, PIK3CA, CHD4, CTNNB1	PTEN, TP53, PIK3CA, CTNNB1, KRAS, CTCF, ARID1A, PIK3R1, FBXW7, ARHGAP35	ERBB2 and ERBB3 mutations are positively selected in normal epithelium, but not in cancer.	^466–470^
Liver	ALB, ACVR2A, HMCN1, ARID2, APC, ESRRG, ARID1A	**ARLD:** JACK1, ARID2, RP1L1, TERT, CDH9 **Cirrhosis:** PKD1, PPARGC1B, KMT2D, ARID1A, STARD9, APOB, ALMS1, ALB, TP53	TERTp, TP53, CTNNB1, ARID1A, ARID2, RPS6KA3, NFE2L2, KRAS, PIK3CA, AXIN1, CDKN2A	Mutations of TERTp mutation occur early in dysplastic nodules and are not detected in healthy or cirrhotic livers.	^136,332,471–473^
Skin (squamous cell)	NOTCH1, FAT1, NOTCH2, TP53, NOTCH3, RBM10, KMT2D	**AKs:** TP53, NOTCH1, FAT1, KIF24, HMCN1, KMT2C, PIK3CA	TP53, CDKN2A, KMT2D, FAT1, NOTCH1, NOTCH2	NOTCH1 and FAT1 mutations are more common in normal tissues than in tumors.	^52,474–476^
Skin (melanocyte)	BRAF, CBL, MAP2K1, NF1, RASA2, ARID2	TERTp, BRAF, NRAS, GNA11, HRAS, CDKN2A, RB1, PPP6C, MAP2K1	BRAF, TERTp, NRAS, CDKN2A, PTEN, ARID1B, ARID1A	BRAF^V600E^ occurs from nevi to tumors. The frequency of CDKN2A mutation increases from intermediate lesions to melanoma.	^57,477^
Breast	PIK3CA, PIK3R1, TP53, PTEN	**BBL with atypia:** PIK3CA, GATA3, PTEN, RUNX1 MAP3K1, CBFB **BBL without atypia:** PIK3CA **DCIS:** CDH1	**Ductal carcinoma:** PIK3CA, GATA3, PTEN, AKT1, CBFB, ATRX, NOTCH2	PIK3CA mutations are found in both normal and proliferative lesions, although they are less common in normal lobules.	^454^

*BE* Barrett’s esophagus, *ESCC* esophageal squamous cell carcinoma, *EAC* esophageal adenocarcinoma, *UC* ulcerative colitis, *ARLD* alcoholic related liver disease, *AA* aplastic anemia, *AD* conventional adenomas, *SSL* sessile serrated lesions, *CRC* colorectal cancer, *AML* acute myeloid leukemia, *CMML* chronic myelomonocytic leukemia, *MF* myelofibrosis, *AKs* actinic keratoses, *MDS* myelodysplastic syndromes, *ADC* adenocarcinoma, *ADH* atypical adenomatous hyperplasia, *AEH* atypical endometrial hyperplasia, *BBL* benign breast lesions, *DCIS* ductal carcinoma in situ

To explore intra-individual heterogeneity, our laboratory analyzed 9 normal organs from the same donors, and found that the liver exhibited the highest mutational burden, significantly surpassing that of other epithelial tissues, whereas the pancreas had the lowest level of mutation burden.^37^ In addition, we compared the mutational signatures across organs and found that aging induced mutagenesis was the most prevalent, although it varied significantly among different tissues. Certain organs, such as livers, were largely influenced by exogenous mutagens. We also spatially reconstructed clonal architecture at sub millimeter resolution, and revealed how clone expansions associate with tissue microstructures, harbored mutations, and environmental factors.^37^ Similar phenomena have also been observed in other studies.^38,40^

Similar to driver mutations in cancer, mutations conferring fitness are positively selected and promote clonal expansion in nonmalignant tissues. Intriguingly, although most driver mutations are classical cancer mutations, they can maintain homeostasis in normal tissues, and exert opposite effects on tumorigenesis.^19^ Furthermore, some mutations are less common in tumors than in normal tissues and have been validated to play a tumor-suppressive role through outcompeting oncogenic clones, exemplified by *NOTCH1* loss of function (LOF) in the esophagus.^41^ In contrast, the frequency of some mutations increases in tumors, like *TP53* in skin, esophageal and endometrial cancers and *PTEN* in endometrial cancer, indicating their contribution to tumor development.^3^ Given that these mutations are generally tolerable in normal tissues, there should exist other factors to further promote their proliferative potential and initiate malignant evolution. To accurately identify additional driver events and the timing they emerge, multiple sampling is required. We recently revealed more detailed genomic changes throughout the entire process of esophageal squamous cell carcinoma (ESCC) formation, using multistep tumorigenesis samples ranging from normal tissue, through low-grade and high-grade intraepithelial neoplasia, to tumors from the same individuals.^42^ We also reconstructed their temporospatial evolutionary dynamics and confirmed that biallelic loss of *TP53* in low-grade intraepithelial neoplasia is one of the earliest steps in initiating malignant transformation, serving as a prerequisite for copy number alterations (CNAs) in oncogenic genes involved in the cell cycle, DNA repair, and apoptosis pathways.^42^ It was also verified in mouse models of esophageal and pancreatic tumorigenesis that *Trp53* loss of heterozygosity (LOH) is a critical step for genomic instability and malignant transformation. Meanwhile, heterozygous *Trp53* mutation can maintain clonality only to a limited extent in normal tissues.^43,44^

#### Copy number alterations and structural variations

Large-scale chromosomal alterations are another widespread form of genetic mutations, encompassing numerical and structural variations and constituting 80–90% of cancer genomes.^45,46^ CNAs comprise aneuploidy, whole-genome duplications (WGDs), and extrachromosomal DNA (ecDNA), while structural variations include genomic catastrophes such as chromothripsis, chromoplexy, and breakage-fusion-bridge cycles. The complex genomic rearrangements have a reciprocal causation with chromosomal instability (CIN), an ongoing state in which cells accelerate the production of aneuploidy, and both of which can converge onto initial chromosome segregation errors.^47,48^ It is speculated that chromosomal alterations occur very early in the evolution of specific cancer types, suggesting their potentially pivotal roles in tumor initiation.^16,49–51^ Indeed, while CNAs and aneuploidy are rarely observed in normal tissues,^19,52–55^ they can be detected in precancerous lesions, albeit at much lower levels than that in fully formed tumors.^56–61^ Furthermore, the levels of CNAs and CIN in precancerous lesions they indicate can serve as indicators of malignant progression.^60,62,63^ ecDNA, a unique form of CNAs, consists of double-stranded circular chromatids, and may serve as a robust driver of tumor genome evolution due to the absence of centromeric sequences and uneven distribution in daughter cells during mitosis.^64^ Notably, ecDNA has been detected early in the progression from high-grade dysplasia in Barrett’s esophagus to esophageal adenocarcinoma (EAC).^64,65^ Their copy number and structural complexity increased along the tumor evolutionary trajectory. Patients who progressed to EAC exhibited higher levels of ecDNA compared to those who did not.^65^

Benefiting from multi-region sampling and single-cell sequencing, ongoing CIN and complex evolutionary processes of CNAs and structural variations can be depicted accurately.^66–69^ Through multi-region sampling of Barrett’s esophagus concurrently containing different states of dysplasia and microscopic EAC foci, it has been reported that the evolution of CNAs during EAC tumorigenesis can be launched ahead of the development of dysplasia. Multigenerational CIN was initiated by mitotic errors and subsequent genomic catastrophes, including WGD, and inactivation of *TP53* played an enabling role in the propagation of CIN, aggravating the accumulation of CNAs.^69^ Recently, signatures of CNAs and CIN have been summarized from pan-cancer studies, encompassing numerous structural and copy number-related biological phenomena, such as WGD, aneuploidy, LOH, homologous recombination deficiency, chromothripsis, and haploidization.^46,70^ It is expected to facilitate integrated analysis of CNAs and structural variations, so as to better elucidate mutational processes and genomic complexity.

Chromosomal abnormalities promote tumorigenesis through their effects on abnormal gene expression, including disruption or loss of tumor suppressors, oncogene amplification, and formation of oncogenic fusion genes.^47,64^ Loss of the 3p arm, harboring tumor suppressor genes such as *VHL*, *PBRM1*, *BAP1*, and *SETD2*, can be an initiating event in clear-cell renal cell carcinoma. An increased frequency of LOH at 9p has been observed from precancerous lesions to cutaneous squamous cell carcinoma (CSCC), possibly driven by loss of tumor suppressive gene *CDKN2A* in this region.^71,72^ Driver fusion genes such as *EML4-ALK* in non-smoker lung adenocarcinoma (LUAD) are speculated to be generated from complex chromosomal rearrangements, including chromothripsis and chromoplexy, and to arise in early years of life.^73^ Specifically, ecDNA can both promote gene amplification and function as mobile enhancers regulating the expression of oncogenes.^74,75^ Nevertheless, it is worth noting that the role of CNAs and structural variations in tumorigenesis are context-dependent.^76^ Complex chromosomal aberrations are likely to exert deleterious cellular effects, inducing senescence, DNA damage, proteotoxicity, essential and toxic gene changes.^77^ However, under specific conditions, aneuploid cells can be preserved, for instance, when WGD occurs ahead, providing extra copies of essential genes to alleviate deleterious alterations.^78^ Furthermore, *TP53* inactivation often occurs earlier to support the occurrence of WGD and clonal expansion.^79,80^ There are also paradoxical immune activation and evasion induced by CIN. Chromosomal mis-segregation generates micronuclei, from which DNA leakage into the cytoplasm can activate the immune system, leading to the clearance of genomic unstable cells via cGAS-STING and type I interferon (IFN) pathway.^81^ At some points, tumor cells develop strategies to overcome the IFN signaling. Simultaneously, the secretome induced by CIN stimulate chronic inflammation and pro-tumorigenic effects.^77,82^

### Epigenetic alterations

The epigenome is another layer of information to encode cell identity and could be passed onto daughter cells. Upon development, natural aging and environmental exposure, there are dynamic changes in DNA and histone covalent modifications that remodel chromatin states and structures, and the heritable epigenetic marks, such as DNA methylation, being referred to as “epimutations”, serve as another important impetus of malignant evolution independent to genetic mutations.^83–85^ Accumulating evidence suggests that clones with aberrantly rewired epigenetic programs show increased tumor susceptibility in morphologically normal tissues.^58,86^ Particularly, age-induced DNA methylation changes are parallel to those seen in malignant states, including increased CpG island methylation and global hypomethylation.^84,87^ During precancerous evolution, epigenomes undergo a stepwise progression, culminating in a high level of intra-tumor heterogeneity in invasive lesions. For instance, a gradual increase of methylation aberrations was observed transitioning from precursors to invasive LUAD.^88^ Actinic keratosis, a precancerous lesion of CSCC, displayed classic cancerous methylome features, with two distinct methylation patterns suggesting different progression pathways to malignancy.^89^ Precancerous colorectal adenomas have also already undergone genome-wide methylation changes and showed preliminary heterogeneity at the adenoma stage.^90^ In specific tumors, such as ependymomas, it seems that epigenetic alterations play a decisive role, with only minimal genetic alterations detected.^91^

Tumor driver events induced by epigenetic reprogramming are presented as overly either restriction or permission states for gene expression, which can induce all hallmarks of cancer.^88^ Highly repressive states induced by DNA hypermethylation lead to gene inactivation, often occurring in tumor suppressor gene related pathways, including DNA repair, cell cycle regulation, and p53 signaling.^92^ Additionally, hypermethylation of promoter CpG islands is frequently observed in lineage-specific transcription factor (TF) sequences that carry bivalent H3K4me3 and H3K27me3 modifications, transforming these previously poised sequences into inactive states that promote dedifferentiation and tumorigenesis.^93,94^ We have confirmed this process in early esophageal tumorigenesis.^95^ Overly permissive states, also known as epigenetic plasticity, can stochastically induce expression of pro-carcinogenic programs. For example, hypomethylation in enhancers and lineage-committed TF regions serves as an important mechanism in leukogenesis,^96,97^ which has already been leveraged by *DNMT3A*^*R822*^ clone in nonmalignant hematopoiesis, leading to chaotic transcriptional phenotypes and increased tumor risks.^96–98^ Another way to induce permissive states and enhance cellular plasticity involves the suppression of Polycomb repressors, such as through the inactivation of histone methyltransferases, as exemplified by early lung tumorigenesis induced by *KMT2D* inactivation.^99,100^ Although DNA hypermethylation mainly induces suppressive states, they can also promote gene expression through dysfunctional chromosomal topology.^101^ Abnormal hypermethylation at cohesin and CCCTC-binding factor (CTCF)-binding sites reduces the binding of insulator protein and formation of insulators, thereby promoting aberrant regulatory interactions like the activation of a constitutive enhancer for the tyrosine kinase gene PDGFRA to upregulate its expression.^101^ An integrative multi-omics atlas of 11 major cancer types indicated that tumor-specific and concurrent epigenetic driver events are associated with cancer transition, with enhancer accessibility playing a more specific role in transition from normal to different types of tumors.^28^ The evidence above suggests that roles of distal regulatory regions and chromatin topology in tumorigenesis warrant further exploration.

Epigenetic alterations and genetic mutations have complex interactions in promoting tumor initiation, with genetic mutations possibly serving as primers to induce epigenetic changes, or epigenetic reprogramming potentiating oncogenic competence of genetic mutations.^102,103^ Genes that encode epigenetic modifiers are common driver mutations in specific cancers and can occur in precancerous stage, such as *TET2*, *DNMT3A* and *ASXL1* in hematologic malignancies,^104,105^ and SWI/SNF chromatin remodeling complexes in solid tumors.^106,107^ Recurrent tumor driver mutations also have capabilities to mediate epigenetic remodeling. For instance, one of the tumor-suppressive roles of p53 is to safeguard epigenetically regulated lineage commitment, and its role in limiting cell fate reprogramming has been proven in several cell types.^108–111^ The oncogenic effects of *Kras* mutations mediated by chromatin remodeling have also been documented.^112^ Conversely, epigenetic priming might precede genetic mutations, rendering cells more susceptible to oncogenic signals, exemplified by aging-related DNA methylation which can activate the Wnt pathway to be more sensitive to *Braf* mutation induced colon transformation.^113,114^ Furthermore, epigenetic abnormalities play a role in accumulating mutations, such as through spontaneous deamination of DNA methylation,^115^ and DNA hypomethylation induced CIN.^63^ In addition, methylated promoters of DNA repair genes underlie a field wherein the colorectal cancer (CRC) with higher rate of mutations arise.^116^ Multi-region single-gland genome, epigenome, and transcriptome profiling of concomitant colorectal adenomas and tumors demonstrated that genetic and epigenetic mutations mutually promoted accumulation of each other. Mutational signature showed that the epigenome alterations induced DNA mutation, while driver mutations were also found in chromatin modifier genes.^117^ However, the functions of chromatin accessible driver genes and genetic driver mutations were independent. Some accessible drivers were devoid of mutations.^117^ Parallel evolution of methylome and genome was also observed in lung tumorigenesis, where global hypomethylation was associated with high mutation burden, CNAs and allelic imbalance, as well as immune infiltration.^88^ Beyond genetic and epigenetic interactions, it is recently reported that chromatin accessibility could also be modified by RNA modification, another regulatory layer for gene expression, being known as epitranscriptome. N^6^-methyladenosine (m^6^A) modifications of RNA are the most common form of mRNA modification, and their roles in regulating transcript stability, translation and localization have been proven to be intricately involved in tumorigenesis.^118^ Recently, specific crosstalk between RNA m^6^A and epigenetic marks, such as histone modifications and DNA methylations, is being unveiled.^119–123^ Our work indicated that m^6^A in super-enhancer RNA is capable of activating YTHDC2 and recruiting H3K4 methyltransferase MLL1 for co-transcriptionally directing H3K4me3 demethylation as well as being accessible to oncogene transcription.^124^ In addition, we also found that m^6^A in RNA could be the cause of DNA demethylation in nearby genomic loci in both normal and cancer cells, which is mediated by RNA m^6^A modification reader FXR1 to recruit DNA dioxygenase TET1.^125^ Altogether, different aspects of chromatin regulation are integrated to regulate cell fate and function. A deeper understanding is warranted to explore their roles and causal relationships.

### Environmental factors

There are diverse environmental and systemic factors that have been epidemiologically confirmed as tumor risk factors, encompassing chemical and radical insults, unhealthy metabolic behaviors, specific pathogen infections, as well as aging. They induce versatile alternations in whole or at local positions, including both induction of genetic and epigenetic alterations in transformed cells and profound impacts on microenvironmental components that predispose to tumor initiation **(**Table 2**)**. Since inflammation is a convergent response to various environmental alterations, we discuss its role in this part at first, which is followed by context-specific mechanisms of other risk factors.

**Table 2:** Gene-environment interactions in tumorigenesis

Tissue	Model	Mutations	Interactions	Refs
**Inflammation**				
Blood	Human	*DNMT3A, TET2, ASXL1, JAK2, SRSF2, PPM1D, TP53*	The cross-sectional study showed expansions of driver mutations in inflammation.	^105,135^
Blood	LPS treated GEMM	*Tet2*^*−/−*^	The *Tet2* mutation upregulates anti-apoptotic lncRNA and modifies the NF-κB inflammatory pathway to prevent HSC impairment. The *Tet2* mutation disrupts intestinal barriers and exacerbate microbial-dependent inflammation.	^142,478,479^
Blood	M. avium infected GEMM	*Dnmt3A*^*−/−*^	Hypermethylation of pro-differentiation factors by *Dnmt3A* LOF attenuates IFN-γ-induced HSC differentiation.	^143^
Blood	TWISTR in zebrafish	*Asxl1*^*−/−*^	Inflammation-resistant genes are upregulated in mutant progenitors. Secretion of IL-1β and TNF-β by mutant daughter macrophages and neutrophils to accelerates inflammation.	^273^
Blood	GEMM treated with IL-1	*Cebpa*^*−/−*^	Mutant cells are resistant to the pro-differentiation effects of chronic IL-1β.	^480^
Blood	GEMM with poly(I:C) injections	*Trp53*^*R172H/+*^	IFN favors the survival of *Trp53* mutated cells while suppressing wild type hematopoiesis in chronic inflammation.	^135^
Colon	Human	*NFKBIZ, ZC3H12A, PIGR, ARID1A, FBXW7*	The cross-sectional study showed the driver mutations were more frequent in UC compared to normal colons.	^137,481^
Intestine	Organoids and GEMMs with DSS induced colitis	*NFKBIZ*^*−/−*^	Being positively selected in inflammatory colons and protecting against CRC.	^137^
Intestine	GEMM with colitis induced by DSS	*Trp53*^*R172H*^	The competitive advantage of *Trp53* mutated ISCs is context-dependent; in inflammatory conditions, they tend to replace wild-type ISCs and initiate tumorigenesis.	^133,134^
Esophagus	GEMM; organoids	*Sox2* overexpression	*Sox2* overexpression promotes the expansion of esophageal basal progenitor cells and inflammation transforms the mutant clones into SCC through the STAT3 pathway.	^138^
Liver	Human	*PKD1, KMT2D, APOB, ALB, ARID1A*	The cross-sectional study linked driver mutations to cirrhosis.	^136,472^
Liver	GEMM with DDC diet induced liver injury	*Pkd1* ^*f/+*^; *Kmt2d* ^*f/+*^; *Arid1a* ^*f/+*^	The mutations promote regeneration after liver injury, independent of tumorigenesis.	^136,482^
Liver	GEMM with DDC diet induced liver injury	*Kras*^*G12D*^*;* *Kras*^*G12D*^, *Trp53*^*−/−*^; *Pten, Trp53*^*f/f*^*;* *Pten*^*f/f*^, *Cdkn2a*^*f/f*^	Liver injury enhances the regenerative capacity of stem cells and increases the risk of cancer induced by oncogenic mutations. Injury and the *Trp53* mutation cooperatively contribute to the reprogramming of hepatocytes into cholangiocytes, initiating intrahepatic cholangiocarcinoma.	^140,483^
Pancreas/ intestine	GEMM; MDCK cell line	*Ras*^*V12*^	Inflammation contributes to cell competition and attenuates the apical extrusion of *Ras-*mutant cells.	^187,484^
Pancreas	GEMM with caerulein induced injury	*Kras*^*G12D*^	The *Kras* mutation hijacks inflammation-induced epigenetic plasticity for dedifferentiation and regeneration, promoting premalignant tumor evolution.	^147–149,157^
**Chemical carcinogens**				
Esophagus	Human	*NOTCH1, TP53, PPM1D, EP300, NOTCH2*	The cross-sectional study linked alcohol consumption and smoking with driver mutations.	^54^
Lung	Human	*ARID1A, ARID2, CHEK2, FAT1, NOTCH1, PTEN, TP53*	The cross-sectional study showed that the driver mutations were more frequent in smokers compared to normal population	^162^
Lung	GEMM exposed to smoke condensate	*Kras*^*V12*^	Epigenetic alterations induced by chronic smoke exposure sensitize cells to *Kras* induced transformation.	^169^
Lung	GEMM exposed to PM2.5	*Egfr*^*L858R*^	PM2.5 promotes IL-1β secretion by macrophages, transforming mutant type 2 epithelial cells into a progenitor-like cell state and promoting tumorigenesis	^170^
Liver	Human	*RP1L1, TERT, JAK1, LRP1B*	The cross-sectional study showed the driver mutations that were more frequent in ARLD compared to that in NARLD.	^332^
Blood	Human	*TET2*	The cross-sectional study linked alcohol with the CHIP.	^105^
Blood	Human	*ASXL1, TET2, DNMT3A*	The cross-sectional study linked smoking with the CHIP.	^485^
Blood	GEMM treated with N-ethyl-N-nitrosourea	*Trp53*^*+/−*^	Mutant p53 is resistant to cytotoxic agents and promote transformation of the therapy related AML.	^486^
Stomach	GEMM with MNU/DCA exposures; organoid	*Trp53*^*−/−*^	Carcinogen exposures and *Trp53* mutation cooperatively activate the WNT pathway and increase renewal properties, promoting gastric premalignancy.	^263^
Pancreas	GEMM exposed to nicotine	*Kras*^*G12V*^	Nicotine enhances activation of Kras and suppresses acinar differentiation regulated by the AKT-ERK-MYC-GATA6, promoting the initiation and progression of pancreatic cancer.	^168^
**UV and other radiation**				
Esophagus	GEMM with low dose ionizing radiation	*Trp53*^*R245W*^	*Trp53*-mutant clones resist radiation-induced oxidative stress and differentiation compared with their wild-type neighbors, gaining competitive advantages for clonal expansion.	^166^
Epidermis	GEMM exposed to UV	*Trp53*^*R245W*^	*Trp53-*mutant progenitor cells tend to proliferation than differentiation under UV and outcompete wild type neighbors, while loss expansive advantages when meeting more competitive mutations induced by UV.	^487^
Skin	Human	*FAT1, NOTCH1, TP53, ARID2, BRAF*	The cross-sectional study showed that sun-exposed skin accumulates more mutations. Chronically sun-exposed melanocytes display a lower mutation burden than intermittently sun-exposed melanocytes.	^52,163,475,477^
Skin	GEMM exposed to UV	*Pten*^*−/−*^, *Braf*^*V600E*^	Melanocyte stem cells with oncogenic mutations are quiescent and can be activated by UV associated inflammation to initiate melanoma transformation.	^488^
Skin	GEMM exposed to UV	*Braf*^*V600E*^	UV exposure induces *Trp53* mutations, leading to the acceleration of *Braf*-driven melanomagenesis.	^489^
Blood	GEMM with total body radiation	*Trp53*^*R248W*^	Mutant p53 interacts with EZH2 to epigenetically regulate the self-renewal property, enabling cells to evade radiation-induced exhaustion.	^167^
Blood	In vitro with UV exposure	*Tet2*^*−/−*^	*TET2* loss in plasmacytoid dendritic cells endows them resistant to UV-induced death, and the accumulation of more oncogenic mutations induced by UV promotes the malignant transformation of blastic plasmacytoid dendritic cell neoplasm.	^165^
**Diets**				
Intestine	GEMM with reduced calorie intake	*Apc*^*−/−*^	Calorie restriction leads to an increased number of stem cells and stronger cell competition, as well as lower retention of mutant tumor-initiating cells.	^490^
Eye imaginal epithelium	Drosophila with diet induced hyperglycemia	*Scrib*^*−/−*^; *Ras*^*G12V*^, *Csk*^*−/−*^	Higher sensitivity of mutant cells to insulin under conditions of hyperglycemia confers selective advantage, triggering tumorigenesis by enhancing protein synthesis, glucose uptake, and inhibiting cell death.	^183,184^
Intestine/ Pancreas	HFD fed GEMM	*Ras*^*V12*^	Enhanced lipid metabolism reverses Warburg-effect-like metabolic changes in EDAC and suppresses the extrusion of *RasV12*-transformed cells, thus promoting tumor initiation.	^187,190^
Pancreas	HFD fed GEMM	*Kras*^*G12D*^	Decreased expression of FGF21 by *Kras*-mutant cells results in severe inflammation, facilitating PanIN progression. The HFD promotes the upregulation of PPARδ in *Kras-*mutant cells, leading to the secretion of CCL2 and the induction of an immunosuppressive microenvironment, driving PanIN progression to PDAC.	^189^
Blood	Human	*TET2*	The cross-sectional study linked elevated circulating apolipoprotein B levels with *TET2* CHIP.	^105^
Blood	Mice with genetic modified hyperglycemia	*Tet2*^*+/–*^	Hyperglycemia-induced inflammation promotes myelogenesis and upregulates the antiapoptotic lncRNA Morrbid expression, establishing a feed-forward loop that initiates AML/MPN.	^491^
**Microbiome**				
Intestine	Human	*APC*	The *APC* mutation is associated with a decrease in probiotics that generate energy, resist inflammation, and maintain lymphocyte and macrophage homeostasis; conversely, bacteria associated with CRC increase in the presence of *APC* mutations.	^492^
Intestine	GEMM and organoids with WNT activation	*Trp53*^*R172H/R270H*^	Mutant p53 serves a tumor-suppressive role by inhibiting the binding of TCF4 to chromatin and preventing the activation of WNT signaling in the proximal gut. This effect is reversed in the distal gut by microbiome metabolism, leading to the oncogenic effects of mutant p53.	^220^
Intestine	*APC*^*Min/+*^ mice with ETBF colonization	*Braf*^*V600E*^	The *Braf* mutation and microbia synergistically induce distinct CpG island DNA hypermethylation and immune signatures, promoting tumorigenesis.	^219^
**Aging and related pathological conditions**				
Esophagus	Human	*PPM1D, TP53, NOTCH1*	The cross-sectional study showed the driver mutations were more frequent in older than in younger individuals.	^54,461^
Skin epidermis	Human	*NOTCH1, FAT1, TP53, NOTCH2, CDKN2A*	The cross-sectional study linked aging with the driver mutations in sun-exposed normal skin.	^163^
Skin epidermis	GEMM	*SmoM2*	Chronic UV exposure and aging decrease the expression of collagen, overcoming the natural resistance to *SmoM2* and give rise to basal cell carcinoma.	^321^
Intestine	Aged organoids; Aged GEMM	*Braf*^*V600E*^; *Braf*^*V637E*^	Aging associated spontaneous methylation sensitizes cells to *Braf* mutation induced transformation, partially due to DNA methylation at WNT pathway.	^113,114^
Liver/ Intestine	MDCK cell line, GEMM and organoids with cellular senescence	*Ras*^*V12*^	The SASP secreted by senescent cells transforms the apical extrusion of mutant cells into EMT-like basal protrusion.	^233^
Mammary	GEMM and MCF10A cell line in the rigid condition	*Her2* activation; *Kras*^*G12V*^	RTK-Ras mutations respond more sensitively to stiff ECM, leading to the transformation of normal cells into precancerous states through the activation of the YAP/TAZ pathway.	^322^

*GEMM* genetically engineered mouse models, *ICC* intrahepatic cholangiocarcinoma, *SCC* squamous cell carcinoma, *IL* interleukins, *PMP* pre-leukemic myeloproliferation, *HFD* high-fat diet, *PDAC* pancreatic ductal adenocarcinoma, *MPN* myeloproliferative neoplasm, *SASP* senescence-associated secretory phenotype, *NARLD* non-alcoholic fatty liver disease, *ETBF* Enterotoxigenic Bacteroides fragilis, *EMT* epithelial-to-mesenchymal transition, *DSS* dextran sulfate sodium, *UC* ulcerative Colitis, *ISCs* intestinal stem cells, *HSCs* hematopoietic stem cells, *ARLD* alcoholic related liver disease, *LOF* loss-of-function, *IFN* interferon, *WT* wild type, *TNF* tumor necrosis factor, *PM2.5* particulate matter measuring ≤2.5 μm, *EDAC* epithelial defense against cancer, *FGF21* fibroblast growth factor 21, *PanIN* pancreatic intraepithelial neoplasms, *CHIP* clonal hematopoiesis of indeterminate potential, *UV* ultraviolet, *AML* acute myeloid leukemia, *TCF4* transcription factor 4, *YAP* yes-associated protein, *TAZ* transcriptional co-activator with PDZ-binding motif, *LPS* lipopolysaccharide, *DDC* dideoxycytidine, *CRC* colorectal cancer

#### Inflammation

Inflammation is a conserved response to potential insults, being involved in tissue repair, regeneration, and homeostasis regulation by stimulating cytokine production and mobilizing innate and adaptive immune systems to remove insults and protect the integrity of the tissue.^126,127^ While acute inflammation aims to solve damage and has tumor-suppressive effects, chronic inflammation caused by unresolved and persistent damage is a well-known tumor risk factor and is considered an enabling hallmark of cancer.^128^ It can be triggered by numerous external stimuli associated with tumors, including chemical carcinogens, radiation, and infections.^129^ Additionally, aberrant autoimmune reactions, such as reflux esophagitis, inflammatory bowel disease, and atrophic gastritis, as well as systemic and subclinical inflammation related to ageing and obesity, can trigger similar pro-tumorigenic effects.^129,130^ The mechanisms by which inflammation is involved in early tumorigenesis include not only oxidative stress and DNA damage, but also priming or releasing the expansion and transformative potential of cells harboring oncogenic mutations.^131,132^ This process can be exemplified by inflammation-stimulated *TP53* mutation clone expansion in colonic and leukemic transformations.^133–135^ Notably, expanding mutant clones in inflammation can play roles independent of tumorigenesis, such as the regeneration role of *ARID1A*, *KMT2D* and *PKD1* in liver injury^136^ and tumor-suppressive *NFKBIZ* mutation in colitis.^137^

It has been widely confirmed that cytokines and growth factors in chronic inflammation play pro-tumorigenic roles, such as interleukin 1 (IL-1), IL-6, transforming growth factor beta (TGF-β), IL-17A, and IL-22, and their functions, which regulate cell survival, proliferation and cell fate determination can be hijacked by cells harboring mutations, activating mitogen-activated protein kinases (MAPK), phosphatidylinositol-3-kinase (PI3K) -AKT, Janus kinase (JAK) -STAT and NF-κB pathways to increase the risk of tumors.^131,132^ Specifically, inflammatory mediators can play a decisive role in early malignant evolution. For example, the cooperation between Sox2 overexpression and inflammation activated STAT3 is capable of inducing ESCC, while in the absence of environmental stimuli, mutations alone may only enhance proliferation without progressing towards tumors.^138^ Liver injury induced dedifferentiation is also a promoter of tumorigenesis, where both mature hepatocytes and cholangiocytes have the potential to give rise to different type of primary liver cancers, comprised of hepatocellular carcinoma and intrahepatic cholangiocarcinoma.^139^ The lineage commitment is dependent on both mutation backgrounds and epigenetic regulations of the injury signaling.^140,141^ Hepatocytes harboring oncogenic mutations induced intrahepatic cholangiocarcinoma upon stimulation of damage-associated molecular patterns (DAMP)-associated cytokines induced by liver cell necroptosis.^141^ By contrast, apoptotic microenvironment promotes transformation of hepatocytes with the same mutation background to hepatocellular carcinoma.^141^ In hematological system, since chronic inflammation leads to stem cell differentiation and exhaustion, mutations conferring resistance to inflammation stress, such as *TET2* and *DNMT3A*, can be positively selected and form clonal hematopoiesis of indeterminate potentials (CHIPs). *Tet2* LOF hematopoietic stem/progenitor cells (HSPCs) upregulated TLR-TRAF6 in response to inflammation, resulting in a shift from the canonical NF-κB pathway to the noncanonical NF-κB pathway, thereby avoiding inflammatory damage to mutated stem cells, and facilitating the *Tet2* mutation-induced progression of myelodysplastic syndrome.^142^ *Dnmt3A* LOF CHIP could also prevent hematopoietic stem cells from terminal differentiation through increasing methylation of IFNγ signaling pathways.^143^

The epigenetic plasticity conferred by inflammation lowers the barriers for malignant transformation. A typical example is pancreatic tumorigenesis initiated from *Kras* mutant acinar cells and promoted by injury and pancreatitis. *Kras* mutation is insufficient for transformation, and injury-induced inflammation is indispensable in the development of pancreatic intraepithelial neoplasms (PanIN) and pancreatic ductal adenocarcinoma (PDAC).^144–146^ Inflammation induces transdifferentiation of acinar cells to ductal cells, which is a reversible process termed as acinar-to-ductal metaplasia (ADM), and can be resolved as tissue regenerates.^145,146^ However, the reprogramming can be co-opted by *Kras* mutations to irreversibly transform the ADM program to PanIN and PDAC programs.^147–149^ Distinct chromatin states between normal regeneration and *Kras* induced tumorigenesis could be mediated by a chromatin reader, bromodomain and extra-terminal family member reader, *BRD4*. The divergence was initiated as early as 48 hours after pancreatic injury induced by caerulein in mouse models.^148^ Besides, another study identified that a precancerous cell subset with ductal identities and oncogenic potential had emerged in ADM, and *Kras* mutation maintained the pro-oncogenic programs, ultimately resulting in PDAC.^149^ It is because inflammation activated AP-1 to dominate the pro-oncogenic transcriptional program, and its key components Junb and Fosl1 could be stabilized by *Kras* mutation.^149^ Similar cooperation between gene and environment was depicted in oncogenic epidermal wound repair, where stress-induced TFs, such as AP-1, ETS2 and STAT3, induced transient lineage infidelity between epidermal stem cells and hair follicle stem cells. In tumorigenesis, stress-TFs were enhanced, resulting in a permanent lineage infidelity and newly activated oncogenic enhancers for malignant transformation, which were divergent from normal regeneration.^150^

An emerging field of study of inflammation-induced epigenetic rewiring is tissue memory, which is an adaptation to recurrent stress and has been identified in various tissues, including skin, lung, intestine and pancreas.^151–157^ In parallel to the immune memory, epithelial cells set long-term memory based on epigenetic modifications they have adopted during injury, which can be partially maintained after the resolution, enabling a more rapid response to a next similar damage.^158^ However, there is a trade-off between tissue long-term adaptation and tumorigenesis that the persistent abnormal epigenetic program primes a field permissive for tumorigenesis. For instance, pancreatic epithelium develops tissue memory of ADM to rapidly instigate a protective program for a secondary pancreatic injury and reduce tissue damage,^157^ which can be enhanced by *Kras* mutations via MAPK constitutive signaling to increase fitness. Nevertheless, *Kras* mutations induces an irreversible ADM reprogramming and increase tumor risk simutaneously.^157^ Similarly, in wound-priming epidermis, there are memory stem cells located in distal intact areas, which are prepared both to respond to another damage adaptively, and to give rise to tumors detrimentally. This is achieved through epigenetic and transcriptional reprogramming and mediated by a long-lasting loss of histone repressive mark H2AK119ub.^159^

#### Chemical and radical insults

Environmental carcinogens are prevalent in nature, derived from air pollution, cigarettes, alcohol, ultraviolet (UV) radiation, etc. These carcinogens promote tumor progression through various mechanisms, including genotoxicity, epigenetic modification, chronic inflammation, immune suppression, oxidative stress, and activation of receptor-mediated signaling pathways.^160,161^

In the tumor initiating stage, chemical and radical carcinogens not only induce mutations and contribute to specific mutational signatures,^54,162^ but also promote clonal expansion of specific mutations. For example, driver mutations of CSCCs, such as *NOTCH, TP53, FAT1* and *FGFR3*, are more prevalent in chronically UV exposed skin than in unexposed healthy skin.^52,163^ Similarly, smoking promotes clonal expansion in the blood, including *ASXL1, DNMT3A*, and *TET2* CHIPs.^105,164^ Intriguingly, the landscape of clone expansion is likely to be reversible. The high mutational burden and driver mutation frequency in the bronchial epithelium decrease after smoking cessation, likely due to the rescue effect of quiescent cell expansion, which was previously protected from tobacco mutagenic insults.^162^

The positively selected mutant clones are expected to exhibit resistance to stress. In sun-exposed skin, plasmacytoid dendritic cells with *Tet2* LOF are protected from UV-induced cell death, providing a reservoir for the accumulation of more oncogenic mutations and subsequent malignant transformation.^165^ Mouse esophageal stem cells harboring *Trp53* mutations are less vulnerable to radiation-induced oxidative stress and replace differentiated wild-type cells for clone expansion.^166^ HSPCs with *Trp53* mutation were also insensitive to radiation-induced differentiation. Mutant p53 bound to enhancer of Zeste homolog 2 (EZH2), a catalytic subunit of Polycomb repressive complex 2 that is responsible for trimethylation of Lys-27 in histone 3 (H3K27me3), thereby promoting the expression of self-renewal program in *Trp53*-mutant CHIP.^167^

In addition to providing a hostile environment, multiple insults can directly activate epithelial cells to induce epigenetic and transcriptional changes, or they can act on immune cells to trigger inflammatory responses, indirectly promoting tumor development. Nicotine activates the AKT-extracellular-regulated kinase (ERK)-MYC pathway via the nicotinic acetylcholine receptor and inhibits the Gata6 promoter, a key regulator of acinar cell differentiation.^168^ This leads to the dedifferentiation of acinar cells and further promotes the activation of *Kras* mutation, thereby facilitating the transformation of *Kras*-mutant ADM and PanIN.^168^ Chronic exposure to cigarette smoke has also proven to induce time-dependent epigenetic changes, which makes bronchial epithelial cells more susceptible to single *Kras* mutation induced tumorigenesis.^169^ Alterations in transformed cells, such as epithelial-to-mesenchymal transition (EMT), anchorage-independent growth, and RAS/MAPK signaling upregulation, are closely associated with gene silencing induced by hypermethylation.^169^ The Epidermal growth factor receptor (*EGFR)* gene mutation is identified as a common driver mutation in healthy lung tissue exposed to environmental particulate matter measuring ≤2.5 μm (PM2.5), and is associated with a higher incidence of LUAD.^170^ Hill et al. showed that PM2.5 induced lung macrophage infiltration and secretion of IL-1β, which mediated the reprogramming of alveolar type (AT) II cells into a progenitor-like state.^170^

#### Metabolic factors

Cellular metabolism is regulated by both intrinsic metabolic properties of the cell and the intake of external nutrients. Tumors modify their metabolic patterns to evade nutrient restraints and fulfill their heightened demands for aberrant growth and proliferation. Alternatively, tumors produce oncogenic metabolites that regulate gene and protein expression to promote tumor progression.^171,172^ Recent findings suggest that metabolic remodeling begins earlier at precancerous stages. In early precancerous lesions of lung squamous cell carcinoma, activities such as fatty acid metabolism, oxidative phosphorylation, and the citric acid cycle are enhanced.^173,174^ These early metabolic changes in tumorigenesis might play a role in driving tumor initiation by interacting with predisposed mutations.^175^ There are two primary mechanisms. One is that mutations drive early metabolic alterations and adaptations. The other is that abnormal metabolic environment facilitates transformation of mutated cells. Classical oncogenic mutations, such as *PIK3CA*, *TP53*, *RAS*, and *MYC*, are all implicated in metabolic regulation by influencing the activity and localization of metabolic enzymes at transcriptional and post-transcriptional levels.^176^ Specifically, they have the potential to recapitulate epigenetic modifications through upregulating expression of metabolic effectors. In the early stage of pancreatic tumorigenesis, mutant *Kras* and loss of *Trp53* enhance acetyl coenzyme A and α-ketoglutarate synthesis, respectively. The metabolites, in turn, epigenetically promote dedifferentiation and PanIN formation.^177,178^ Additionally, *Kras* mutations promote metabolic remodeling via post-translational modification of metabolic enzymes. They suppress ubiquitylation and degradation of branched-chain amino acid transaminase 2, an enzyme essential for the catabolism of branched-chain amino acids and mitochondrial respiration, thereby contributing to the progression of PanINs.^179^ Apart from recurrent cancer mutations, mutations in genes encoding metabolic enzymes, including succinate dehydrogenase, fumarate hydratase, and isocitrate dehydrogenase 1 or 2, have the capability to accumulate oncometabolites, disrupting dioxygenases and their epigenetic regulatory functions.^171^ The isocitrate dehydrogenase mutation induced oncometabolite, (R)-2-hydroxyglutarate, was confirmed to promote the early tumorigenesis of acute myeloid leukemia (AML) and gliomas through the inhibition of histone lysine demethylases 5.^180^

In addition to mutation-driven metabolic remodeling, unhealthy systemic metabolic status, including high-fat and high-carbohydrate diets, and metabolic diseases they induce, such as obesity and type 2 diabetes mellitus, can increase the risk of tumors.^181,182^ Obesity triggers several pathological processes associated with tumor development, including hyperglycemia-related insulin resistance, abnormal hormone secretion, inflammation and dysregulation of lipid metabolism. Under physical conditions, insulin signaling systematically senses blood glucose levels and promotes proliferation and anabolic metabolism. In the presence of obesity, insulin resistance in metabolic tissues leads to hyperglycemia and hyperinsulinemia, while tumor cells develop strategies to maintain their sensitivity to insulin-induced proliferative signaling.^181^ Transformed mutant cells can adopt similar strategies, utilizing the proliferative signaling and gaining competitive advantages.^183,184^ Furthermore, hyperglycemia induced by both glucose and fructose consumption enhances tumorigenesis by accelerating glycolysis and de novo lipogenesis.^185^ Recently, glucose was reported to act as a signaling molecule, directly binding to and activating NSUN2, thereby activating NSUN2-TREX2 signaling. This led to inhibition of dsDNA accumulation, subsequent cGAS/STING pathway activation, and immune activation.^186^ In terms of the high-fat diet (HFD), Sasak et al. found that it disrupted cell competition outcomes by enhancing lipid metabolism.^187^ In normal epithelium, the apical extrusion of Ras^V12^ transformed cells could be mediated by Warburg-like effects and damage to mitochondrial membrane potential. However, HFD increased the levels of free fatty acids and promoted their metabolic transformation to acetyl coenzyme A, which played a role in restoring the mitochondrial membrane potential and inhibited the clearance of Ras^V12^ cells.^187,188^ Furthermore, inflammation and immune responses play crucial roles in linking the pathological processes of obesity to tumorigenesis.^189^ Pancreatic *Kras* mutation can downregulate peroxisome proliferator-activated receptor (PPAR)-γ, exacerbate inflammation and further promote the formation of PanIN, mediated by fibroblast growth factor 21, which is an endocrine regulator for metabolic homeostasis.^189^ HFD also promotes the activation of PPAR-δ and the secretion of CCL2 in *Kras*-mutant pancreatic cells. Consequently, immunosuppressive cells are recruited, promoting the transformation from PanIN to PDAC.^190^ Additionally, the mechanisms by which HFD promotes tumorigenesis are also related to microbial dysbiosis. Alterations in gut microbiota and metabolites are crucial for HFD-associated colorectal tumorigenesis, inducing cell proliferation, impairing gut barriers, and promoting oncogenic gene expression.^191^

#### Microbiome

The human body harbors diverse microbiome communities that interact with the host in complex ways.^192^ Dysbiosis has been implicated in the development of numerous diseases, including cancer.^128^ The tumorigenic effects of specific microorganisms have been well-established across several types of tumors. The World Health Organization has classified several microorganisms as Group 1 carcinogens, including *Helicobacter pylori* (*H. pylori*), Epstein-Barr virus (EBV), human papillomavirus, hepatitis B virus (HBV), and hepatitis C virus (HCV).^193^ Excepted for the classic oncogenic pathogens, many other microorganisms have also been discovered to be associated with tumors,^194^ and several large-scale pan-cancer studies have revealed the presence of microbes in almost all types of tumors.^195–197^ For example, *Fusobacterium nucleatum*, polyketide synthase-positive(*pks*^+^) *Escherichia coli* (*E. coli*), and enterotoxigenic *Bacteroides fragilis* (ETBF) are associated with the occurrence of CRC.^198–200^ Furthermore, it has recently been discovered that microbiome alterations emerge in early precancerous stages of CRC, indicating their promoting role in early tumorigenesis.^201–203^ In other tumor types, there are also some cues that microbiota is involved in tumor formation, such as *Streptococcus anginosus* (*S. anginosus*) in gastric cancer,^204^ *Acidovorax* species in lung squamous cell carcinoma,^205^ and *Bacteroides fragilis* in breast cancer.^206^

The microbiome plays a crucial role in tumorigenesis through various mechanisms, including physical binding or secretion of metabolites and toxins, which lead to genotoxicity and epigenomic abnormalities, activation of signaling pathways, and modulation of the immune system and inflammatory responses.^207,208^ One of the most well-known examples of genotoxicity is the integration of the HBV genome into the host liver cell genome, which results in genetic mutations and chromosomal abnormalities that promote liver cancer.^209,210^ HBV DNA most commonly integrates into the telomerase reverse transcriptase (*TERT*) promoter region, disrupting the tight suppression of *TERT* transcription and leading to abnormal liver cell proliferation.^211–213^ Furthermore, when HBV inserts into the human cyclin A gene, it generates novel tumor-specific chimeric proteins with oncogenic functions.^214–216^ Pathogenic *E. coli* also promotes tumorigenesis through genomic alterations. The toxin colibactin, secreted by *pks*^+^ *E. coli*, causes interchain crosslinking and double-strand DNA breaks, leading to gene mutations and tumorigenesis.^217,218^

In addition to genetic mutations, it has been reported that bacteria significantly contribute to epigenetic alterations.^219,220^ The human commensal bacterium ETBF could promote distal colonic tumorigenesis in the *Apc*^*MinΔ716/+*^ mouse model. When another *Braf*^*V600E*^ was induced, new tumors emerged in the midproximal colon, which exhibited similar phenotypes to human *BRAF*-mutant serrated-like tumors. The colonization of ETBF and *Braf* mutation synergistically increased the levels of CpG islands DNA methylation and induced characteristic immunophenotypic alterations, including IFN pathway activation, and myeloid-derived suppressor cells and CD8^+^ T cell infiltration.^219^ Furthermore, the microbiota can exert an epigenetic modulation role by influencing the oncogenic effects of mutant proteins. *Trp53* mutation plays context-specific roles in intestinal tumorigenesis, promoting tumorigenesis in the distal gut while suppressing tumors in the proximal gut.^220^ The tumor suppressive effect was achieved through disrupting the binding of T cell factor 4 to chromatin and repression of the WNT signaling. A high density of microorganisms in the distal gut, along with their metabolite gallic acid, has the potential to reverse the protective role of mutant p53 and activate the oncogenic WNT pathway. The administration of antibiotics effectively reduced WNT activation and cell proliferation.^220^ Furthermore, Fu et al. recently discovered that *S. anginosus* promoted the tumorigenesis of *H. pylori*-negative gastric cancer through direct interactions.^221^ The surface protein of *S. anginosus*, TMPC, could activate gastric epithelial cell receptor ANXA2, enabling colonization of *S. anginosus* in gastric mucosa and activation of MAPK pathway.^221^ As a result, *S. anginosus* damaged the gastric barrier function, promoted cell proliferation, and inhibited apoptosis of epithelial cells, and ultimately induced gastric cancers.^221^

Microbes can also play a pro-tumoral role by regulating the immune microenvironment. The microbiome in pancreatic cancer selectively activates Toll-like receptors in monocytes, which in turn drives immune suppression by inducing T-cell anergy, ultimately fostering tumorigenesis.^222^ Fungi migrating from the intestine to the pancreas also experience fungal dysbiosis. They activate the mannose-binding lectin-complement cascade reaction to accelerate PDAC formation.^223^ On the contrary, some microorganisms play roles in inhibiting immunosuppression and tumor formation.^224,225^ *Ruminococcus gnavus* and *Blautia producta*, belonging to Lachnospiraceae family, could inhibit the growth of colon tumors by degrading dissolved glycerophospholipids, suppressing their immunosuppressive function, and maintaining the immune surveillance function of CD8 T cells.^224^ Similarly, during the occurrence of CRC, the urea cycle is activated because of the absence of beneficial bacteria with ureolytic capacity. The accumulation of urea could induce macrophages to polarize towards a pro-tumorigenic phenotype, characterized by polyamine accumulation, thereby promoting the tumorigenesis of CRC.^225^ Altogether, the complex crosstalk between the microbiome and their hosts in tumorigenesis involves both tumor cells and their microenvironmental cells, inducing changes at genetic, epigenetic, transcriptional, and metabolic levels, which warrants further exploration.

#### Aging

Aging is considered the primary risk factor for tumorigenesis.^226^ There are systemic and local changes that overlap with that in tumors, including genomic instability, epigenetic alterations, inflammatory responses, and dysbiosis,^227^ which may have already played a role as early as in tumor initiating stages. Abnormal epigenetic alterations associated with aging underlie mutation-induced tumorigenesis. In mouse-derived organoids, aging-like spontaneous methylation of DNA promoter CpG-island induced colon more susceptible to the *Braf*^*V600E*^-driven proximal colon tumorigenesis by activating Wnt signaling.^114^ However, there are some aging hallmarks, including telomere attrition, decreased stem cell plasticity, and cellular senescence-associated cell cycle arrest, possessing tumor-suppressive properties.^227^ Tumor-initiating cells always evade these tumor-suppressive mechanisms through mutations, such as inactivating mutations in *TP53, CDKN2A*, and *CIP1*.^228^ Moreover, mutations in the promoter of TERT, which allow for the maintenance of telomeres, are one of the most common driver mutations in a variety of tumors, and can be detected even in cirrhotic regenerative nodules, preventing cellular senescence and cell-cycle arrest, and thereby enhancing the proliferative potential of the transformed cells.^213,229^

In addition to transformed cells, various microenvironmental cells, including fibroblasts, immune cells, and endothelial cells, generally exhibit an increased rate of senescence.^230,231^ This is accompanied by the secretion of a large quantity of senescence-associated secretory phenotype, including various cytokines, growth factors, enzymes, and extracellular matrix (ECM). Although senescence-associated secretory phenotypes promote the clearance of senescent cells by activating the immune system in youth, it exerts immunosuppressive, pro-inflammatory, and pro-fibrotic effects in aging and chronic inflammation, contributing to tumorigenesis by directly targeting tumor cells or indirectly remodeling the microenvironment.^232^ In cell competition, hepatocyte growth factor, a component of the senescence-associated secretory phenotype secreted by fibroblasts, was confirmed to inhibit *Ras*^*V12*^ cell elimination by inducing their EMT and transformation from apical to basal extrusion.^233^ Furthermore, the senescence and dysfunction of immune cells can lead to immunosuppression, possibly further increasing the risk of cancer.^234^ Clearance of senescent macrophages was shown to reduce tumor burden and intercept non-small cell lung cancer at early and intermediate tumor stages by promoting immune surveillance in a *Kras*-driven lung cancer model.^235^

## Key processes required for early tumorigenesis

**Fig. 3:**
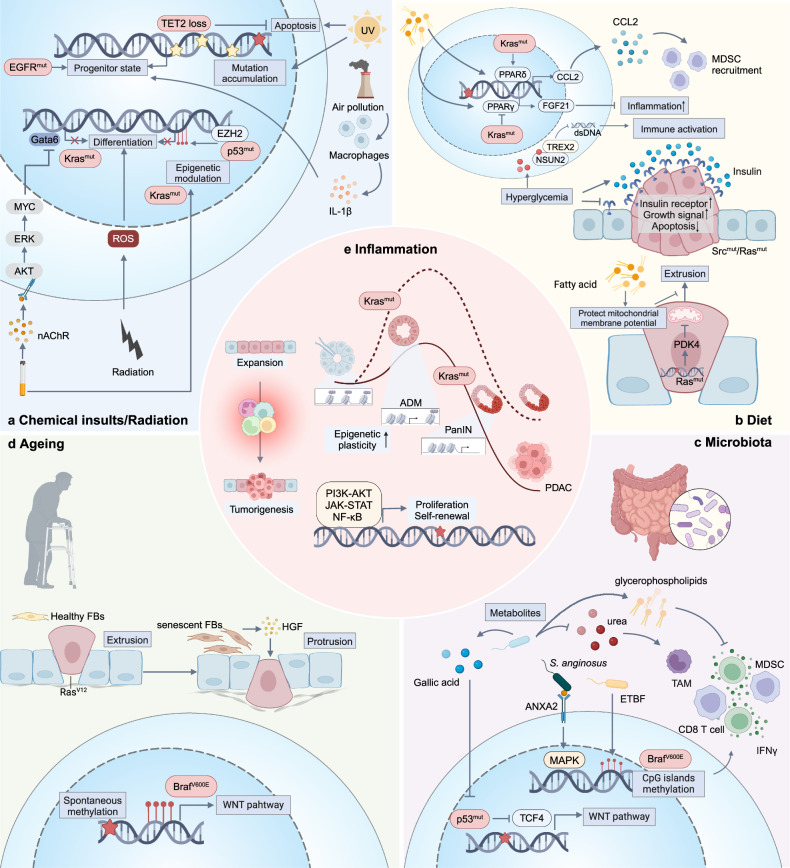
Interactions between oncogenic driver events. **a** In addition to genotoxicity, chemical and radical insults can induce cell injury, differentiation, and apoptosis. Oncogenic mutations that can confer resistance to such injuries provide proliferative advantages. On the other hand, the insults stimulate proliferative and self-renewal pathways by transcriptional and epigenetic regulation. Immune cells can also be activated to regulate transformed cell fate and promote tumorigenesis. **b** Unhealthy diet patterns induce hyperglycemia and hyperinsulinemia, and further cause differential response to insulin signals, which can facilitate cells harboring *Src* or *Ras* mutation in gaining competitive advantages and promote tumorigenesis. High levels of fatty acids also promote retention of *Ras*-mutant cells in cell competition by metabolism remodeling and mitochondrial membrane potential restoration. In addition, fatty acid and glucose participate in tumorigenesis as signaling molecules by modulating immune response and inflammation. **c** Microbiota interacts with transformed cells to affect host DNA methylation, transcription, metabolism and immune microenvironment to have an influence on malignant transformation. **d** Aging induces senescent stromal cells to secrete SASPs, which can reverse the outcome of cell competition and promote EMT of the mutant cell. Aging also cause spontaneous methylation, further promoting mutation-driven tumorigenesis. **e** The pathological processes mentioned above can converge at inflammation, which releases tumorigenic potential of expansive clones by activating oncogenic pathways and increases epigenetic plasticity. For instance, in pancreatic inflammation induced plastic state, ADM, *Kras-*mutant cells are more likely to transform to malignant status, while in the absence of inflammation, *Kras* can only induce PanIN without progression to PDAC. EMT epithelial-to-mesenchyma transition, ROS reactive oxygen species, nAChR nicotinic acetylcholine receptor, MDSC myeloid-derived suppressor cell, PDK pyruvate dehydrogenase kinase, TCF4 T cell factor 4, HGF hepatocyte growth factor, TET2 tet methylcytosine dioxygenase 2, EGFR Epidermal growth factor receptor, UV ultraviolet, Gata6 GATA Binding Protein 6, EZH2 enhancer of zeste homolog 2, PPAR-δ peroxisome proliferator-activated receptor-delta, FGF21 fibroblast growth factor 21, CCL2 PDK4, pyruvate dehydrogenase kinase 4, ADM acinar-to-ductal metaplasia, PanIN pancreatic intraepithelial neoplasms, PDAC pancreatic ductal adenocarcinoma, ETBF enterotoxigenic *Bacteroides fragilis*, Created with BioRender.com

The identities of transformed cells are the result of the combined influence of intrinsic genetic and epigenetic profiles and external signaling. These factors collectively activate oncogenic pathways and remodel the microenvironment (Fig. 3). Consequently, there are not only cell-autonomous alterations that override cellular quality control mechanisms, enabling the gradual acquisition of hallmarks of cancer, but also adaptations to the extrinsic stress from their surrounding healthy counterparts, microenvironmental components, and tissue architecture. In addition, transformed cells actively reshape the external factors to be tailored for their oncogenic identities.

### Cell-autonomous processes

**Fig. 4:**
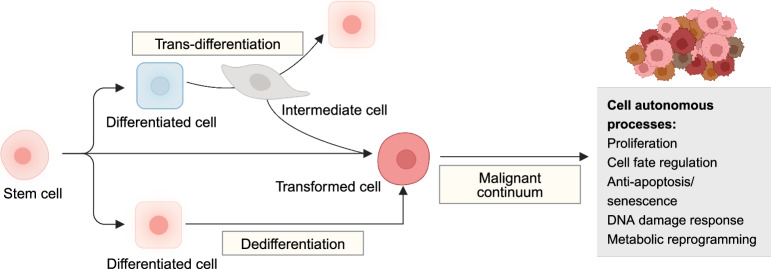
Cell-autonomous processes in tumorigenesis. After acquiring genetic and epigenetic mutations, transformed cells enter a malignant continuum where they reprogram their developmental pathways, allowing gradually gains of uncontrollable self-renewal capabilities and aberrant differentiation potential, primarily through three mechanisms: originating from stem cells, dedifferentiating from lineage-committed cells, and hijacking intermediate states during trans-differentiation. Created with BioRender.com

Cells in normal tissues are hierarchically organized to restrain tumorigenesis. The initiating transformed cells must reprogram their cell fates, so as to gain uncontrollable self-renewal abilities and aberrant differentiation.^2^ There are mainly three ways, encompassing activation of unlimited proliferative potential in stem cells, dedifferentiation of lineage-committed and differentiating cells, as well as leveraging intermediate states during trans-differentiation as the precursor of cancer (Fig. 4).

It is believed that stem or early progenitor cells are more likely to achieve malignant transformation, based on their inherent self-renewal capacity and longevity.^236–238^ On one hand, stem cells can accumulate more genetic mutations and epigenetic alterations necessary for tumor formation.^239^ On the other hand, stem cells and early progenitor cells exhibit high levels of cellular plasticity and are highly susceptible to fate transition.^240^ In the developmental hierarchy of melanocytes, progenitor stages, including neural crest and melanoblasts, are susceptible to transformation by *BRAF*^*V600E*^ and additional mutations, while differentiated melanocytes resist these cancerous signals.^241^ The difference is induced by ATPase family AAA domain-containing 2 (ATAD2) in neural crest and melanoblasts, which regulates chromatin accessibility.^241^ This enables TFs including SOX10 and MYC to form complexes with ATAD2, initiating the expression of downstream neural crest genes and oncogenic MAPK pathway genes, respectively.^240^

In addition to stem cells, there is accumulating evidence suggesting that committed cells are also able to give rise to cancer, specifically after undergoing dedifferentiation into stem-like cells upon oncogenic mutations or environmental stimulation.^242–244^ For instance, melanoma induced by *Braf*^*V600E*^ and *Pten* loss can be originated from mature, pigment-producing melanocytes located in the interfollicular regions of mouse tails, which experienced transcriptional reprogramming and dedifferentiation prior to invasion.^245^ Consistently, Kaufman et al. identified the fate change during melanoma initiation in a *Braf*^*V600E*^ and *Tp53* loss zebrafish model. Re-expression of neural crest progenitor program in melanoma, characterized by embryonically expressed gene *Crestin*, was driven by neural crest progenitor transcriptional factors, such as SOX10.^246^ Another example where dedifferentiation is implicated in tumor initiation is observed in mammary epithelium. *Pik3ca* mutation in lineage-restricted mammary basal and luminal cells can both induce multipotent stem-like cells, which is followed by development of tumor heterogeneity and multilineage mammary tumors.^247^ Luminal progenitor cells derived from *BRCA1* basal-like breast cancers have also been confirmed to undergo dedifferentiation.^248,249^ Mechanically, MYC plays a central role in the reprogramming of the lineage-specific cells. It inhibits mammary luminal-specific TFs, leading to the decommissioning of enhancers that disrupts their original transcriptional program. Additionally, MYC activates de novo enhancers and activates oncogenic pathways, such as the WNT pathway, which supports stem cell features and predisposes luminal epithelial cells to tumor initiation.^250^

Trans-differentiation is a common physiological response to injury, converting cells that are initially committed to one differentiation fate into an entirely different direction, either directly or through a stem or progenitor cell intermediate. The process can be implicated in tumor initiation, as exemplified in lung tumorigenesis that hijacks repair and regeneration programs. Many types of lung epithelial cells are highly plastic, and are capable of abandoning their cell fidelity to differentiate into each other upon injury, such as the transformation of club cells into AT2 cells, and AT2 cells into AT1 cells, which can be leveraged to promote tumorigenesis.^111,251–254^ Specifically, the intermediary state during these transformations is likely to be the key progenitor giving rise to tumors. For instance, KRT8 intermediate cells, which transition between AT2 and AT1 cells, have been identified in normal lung tissues adjacent to LUAD lesions.^253^ The KRT8 cells expand in precancerous and cancerous stages and are implicated in tobacco-associated *KRAS*-mutant LUAD,^253^ marked as reduced differentiation, enhanced plasticity and harboring *KRAS* driver mutations.^253^ The high-plasticity cells can also play a role in later progression and development of tumor heterogeneity. In a mouse model of LUAD tumorigenesis originating from *Kras*^*G12D*^ mutation and *Trp53* loss in AT2 cells, a subset of transitional and high-plasticity cells emerging from adenomas was computationally predicted to drive cellular heterogeneity.^254^Although they are distinct from stem cells, they exhibit high growth and differentiation potential and play a transitional role in giving rise to the most heterogeneous cancer cell identities, which are indispensable for LUAD progression^254^ Other classic cases include pancreatic and epidemic injury, where lineage infidelity and epigenetic reprogramming at intermediate stages can be exploited by oncogenic mutations to activate malignant programs.^147,150,255,256^ Specifically, it is suggested that EMT is a drastic state of plasticity, and its intermediate state also exists, which endows cells with the highest capacity of invasion and metastasis.^257,258^ Recent evidence indicates that the EMT can occur at a very early stage of tumorigenesis.^259,260^ In squamous cell carcinomas induced by *FAT1* LOF, the mutation triggers both a mesenchymal state mediated by YAP1-ZEB1 and a sustained epithelium state through EZH2 inactivation and SOX2 expression, illustrating a hybrid EMT phenotype with enhanced stemness and increased metastatic potential.^260^

Recently, a series of studies analyzing different stages of precancerous samples across various tumor types at single-cell resolution have demonstrated dynamic evolutionary trajectories preceding tumor formation, revealing a continuum of changes that lead to acquisition of hallmarks of cancers, including cell cycle, cell fate regulation, and metabolic reprogramming^128^ (Table 3). For example, through single-cell multi-omics analysis of HSPCs from patients with myeloproliferative neoplasm, a convergent genomic evolutionary pattern of a double-hit *TP53* mutation in hematopoietic stem cells was identified, and based on this trajectory, pre-leukemia stem cells ultimately progressing to secondary AML were found to undergo differentiation arrest prior to *TP53* mutation occurrence, and the subsequent P53 mutant clones could be selected by inflammation, leading to clonal expansion.^135^ Conventional colon adenomas can be traced back to originating from colonic stem cell (CSC). Throughout the progression from normal stem cells to adenomas and then to colon cancer, there is a gradual change in gene expression and chromatin accessibility, including upregulation of stem-like programs and increased antioxidative stress capability.^261,262^ On the other hand, premalignant phenotypes induced by intrinsic and environmental drivers have been explicitly depicted in preclinical models. In mouse models and organoids, gastric premalignancies resulting from *Trp53* mutations and exposures relevant to the disease have demonstrated the acquisition of renewal properties, activation of the WNT pathway independent of exogenous WNT ligands, and the abilities to overcome cell cycle distress and DNA damage stress.^263^ Similarly, progenitors of pancreatic tumorigenesis, induced by *Kras* mutations and inflammation, are characterized as gaining proliferative potential, with activation of cell cycle genes and other pathways.^150^ Furthermore, in colorectal cancer originating from CSCs, CSCs are fixed predominantly on a highly proliferative phenoscape, whereas there is a continuous differentiation phenoscape that spans revival CSCs to proliferative CSCs under normal condition.^264^ YAP signaling regulates polarization of revival stem cells, which can be activated by fibroblast derived TGF-β, while *APC* loss and *KRAS*^*G12D*^ mutation collaboratively activate MAPK-PI3K signaling, trapping CSCs in the cancerous proliferative fate.^264^ Compared to ECM signaling, the intrinsic mutations exert a more dominant effect in regulating the stem cell fates.^264^ The evidence above also suggests that the regulation of malignant transformation involves the interplay between intrinsic cellular factors and microenvironmental factors, which needs to be evaluated in a tissue-specific context.

**Table 3:** Evolution of transformed cells and microenvironment in tumorigenesis

Components	Tissue	N	Early evolution	Refs
Transformed cells	colon	128^a^	Conventional adenomas originate from WNT-driven expansion of stem cells, while SSLs develop from lineage-committed cells. Downregulation of CDX2 in serrated specific cells supports a loss of regional identity and emergence of a fetal gene expression signature.	^262^
	colon	81^a^	Stem-like cells form a malignancy continuum from early and late polyps to CRC, along which the WNT signal increases and the glutathione peroxidase increases to reduce the oxidative stress.	^261^
	colon	72^a^	Proliferation and DNA damage repair increase, while mitochondrial function and lipid metabolism decrease from normal tissue to CRC.	^279^
	skin	52	Sequential dedifferentiation that recapitulates the ordered cascade of differentiation in reverse is predominant in melanoma progression	^493^
	esophagus	43	Differentiated BE cells decrease during the transition from BE to EAC while the undifferentiated BE phenotype is maintained; transcription factor HNF4A is activated in differentiated BE cells and MYC is activated in undifferentiated BE cells.	^494^
	esophagus	29	Quiescent progenitor, cycling, mucosal defense, and terminal differentiation programs decrease expression, while hypoxia-related stress, reactive oxygen species-related stress, deoxidation, and antigen presenting programs increase from normal to LGIN-HGIN-ESCC.	^307^
	blood	31	Differentiation defects, distinct stemness, self-renewal and quiescence signatures of *TP53* wild-type pre-LSCs HSCs in sAML are distinct from HSCs in healthy and myelofibrosis samples. Chronic inflammation enhances the fitness of *TP53*-mutant cells for clonal expansion.	^135^
	lung	25	Energy metabolism and ribosome synthesis programs are upregulated in AT2-like cells that emerge during AAH, which is diverged from normal AT2 cells and gain stemness as LUAD progresses.	^62^
	lung	9	Differentiation decreases from normal to cancer cells.	^495^
	stomach	25	mTOR signaling, RAS pathway, and VEGF signaling, functioning in IGC, are highly enriched in tumor cells.	^496^
	oral cavity	9	EMT, mTORC1, and FRA pathways increase during the OSCC initiation.	^414^
Immune cells	colon	128^a^	The cytotoxic immune response is more significant in serrated polyps compared to conventional adenomas, and the distinction persists in advanced tumors.	^262^
	colon	72^a^	The relative abundance of plasma cells, B cells, CD8 + T cells, CD4 + T cells, Treg cells, γδ T cells and NK cells decrease, and that of macrophages increase from normal tissue to carcinoma.	^279^
	colon	81^a^	The number of Tregs increases from polyps to carcinoma; Exhausted T cells only appear in CRC.	^261^
	stomach	43	The number of IgA^+^ plasma cells increases in chronic atrophic gastritis and intestinal metaplasia, and declines in GAC; Myeloid cells transition from immune-activating to immune-suppressive properties as they progress from intestinal metaplasia to GAC.	^497^
	lung	62	AIS lesions that can regress to normal have more infiltrating immune cells, while progressive lesions have developed immune escape mechanisms in precancerous stages.	^281^
	lung	53	Immunosuppression initiates at the preneoplastic stage, characterized by a reduction in T cell infiltration and clonality, and an increase in Tregs.	^280^
	lung	41	T cell infiltration is associated with mutation-induced neoantigens, alongside PD-L1 upregulation in T cells from AAH to ADC.	^415^
	breast	35^a^	DCISs have active immune responses while transforming to suppressive one in IDCs.	^498^
	bone marrow	65	Mature B cells, NK cells, and CD14+ monocytes decrease and CD16+ monocytes, tumor-associated macrophages increase from normal to smoldering MM.	^452^
	bone marrow	32	The number of CD8 + T cells decreases in smoldering MM and MM compared with healthy and MGUS.	^499^
	bone marrow	14	The proportion of T cells decreases from smoldering MM to MM, while monocytes contribute to the largest proportion in primary MM.	^500^
	pancreas	30	Myeloid cells and CD4 + T cells are more enriched in ADM and PanIN compared to normal pancreas.	^501^
	esophagus	29	Changes in immune cells are late events in ESCC tumorigenesis, and the proportion of CD8 + T cells is similar from normal to LGIN and HGIN while increasing in ESCC.	^307^
	esophagus	19	Macrophages increase from esophageal squamous precancerous lesions to ESCC but M2 macrophage cells and Treg cells decrease in ESCC compared to the precancerous lesions stages.	^502^
Fibroblast /CAFs	colon	81^a^	Pre-CAFs are enriched in polyps and express RUNX1 to epigenetically regulate CAF programs.	^261^
	colon	72^a^	The relative abundance of fibroblasts increases from normal tissue to carcinoma.	^279^
	breast	79	Normal fibroblasts transit to CAFs from primary DCIS to later invasive breast cancer.	^366^
	stomach	43	myCAFs are dominant in intestinal metaplasia and enriched in SDC2 expression, which is associated with aggressive progression and poor prognosis.	^497^
	stomach	5	The PDGFRα+ fibroblasts expand in metaplasia and cancer compared with normal.	^503^
	stomach	24	CAFs occur from the precancerous state, and the number increases in the malignancy; The iCAFs exhibits pro-stemness property and may promote diffuse type GAC tumorigenesis.	^496^
	pancreas	30	PanINs are surrounded by fibroblasts and the diversity of fibroblasts gradually increase from normal to tumors.	^501^
	pancreas	6	Fibroblasts activation and myCAF subtype transformation occur from LGD-IPMNs to HGD-IPMNs.	^504^
	esophagus	29	CAFs are activated in the HGIN and ESCC, rather than in normal and LGIN tissues.	^307^
	skin	14	Fibroblasts have not yet been fully activated in actinic keratosis; myCAFs increased more than iCAFs in tumorigenesis	^505^
	oral cavity	9	CAFs increase from normal epithelium to dysplasia and tumors, among which myCAFs are the dominant subtype.	^414^
Micro-organisms	colon	616	*Fusobacterium nucleatum* spp. elevates continually from intramucosal carcinoma to advanced tumors. *Atopobium parvulum* and *Actinomyces odontolyticus* only increase from LGD to intramucosal carcinoma. Fecal metabolites, including BCAAs and phenylalanine, increase from intramucosal carcinoma, while bile acids increase from LGD.	^201^
	colon	431	A panel of gut microbiome-associated serum metabolites alters from normal, through adenoma to CRC.	^202^
	colon	386	Integration of fecal metabolites and microbiome analysis can distinguish CRCs with adenomas and normal tissues.	^203^

*SSL* sessile serrated lesion, *CRC* colorectal cancer, *BE* barrett’s esophagus, *EAC* esophageal adenocarcinoma, *LGIN* low grade intraepithelial neoplasia, *HGIN* high grade intraepithelial neoplasia, *ESCC* esophageal squamous cell carcinoma, *Pre-LSCs* pre-leukemic stem cells, *HSCs* hematopoietic stem cells, *LGD* low-grade dysplasia, *HGD* high-grade dysplasia, *AT2* alveolar type 2 cell, *sAML* secondary acute myeloid leukemia, *IPMNs* intraductal papillary mucinous neoplasms, *OSCC* oral squamous cell carcinoma, *OLK* oral leukoplakia, *PanIN* pancreatic intraepithelial neoplasia, *CAFs* cancer-associated fibroblasts, *MDSCs* myeloid-derived suppressor cells, *AAH* atypical adenomatous hyperplasia, *AIS* adenocarcinoma in situ, *ADC* adenocarcinoma, *IPNs* indeterminate pulmonary nodules, *DCIS* ductal carcinoma in situ, *IDCs* invasive ductal carcinomas, *SDC2* Syndecan 2, *GAC* gastric adenocarcinoma, *MM* multiple myeloma, *MGUS* monoclonal gammopathy of undetermined significance, *NK* natural killer, *ADM* acinar-to-ductal metaplasia, *BCAAs* branched-chain amino acids, *ECM* extracellular matrix, *Treg* regulatory T cells, *myCAF* myofibroblastic CAF, *LUAD* lung adenocarcinoma, *IGC* intestinal-type gastric cancer, *EMT* epithelial-mesenchymal transition, *iCAF* inflammatory CAF

^a^Numbers of samples, the others are number of individuals

### Clonal expansion by cell competition

Multicellular organisms develop surveillance mechanisms that compare cellular fitness with neighboring cells to preserve the most robust populations in environments with limited space and nutrients, a process termed ‘cell competition’. In epithelial tissues, mutant cells that alter fitness often become the losers and are eliminated by neighboring wild-type cells. Therefore, the process is an important tumor-suppressive mechanism, referred to as ‘epithelial defense against cancer’.^265^ However, in some cases, mutations can endow cells with ‘winner’ properties, allowing them to eliminate surrounding normal cells and gaining space for clonal expansion and tumor development, which is called ‘supercompetitor’.^266^

**Fig. 5:**
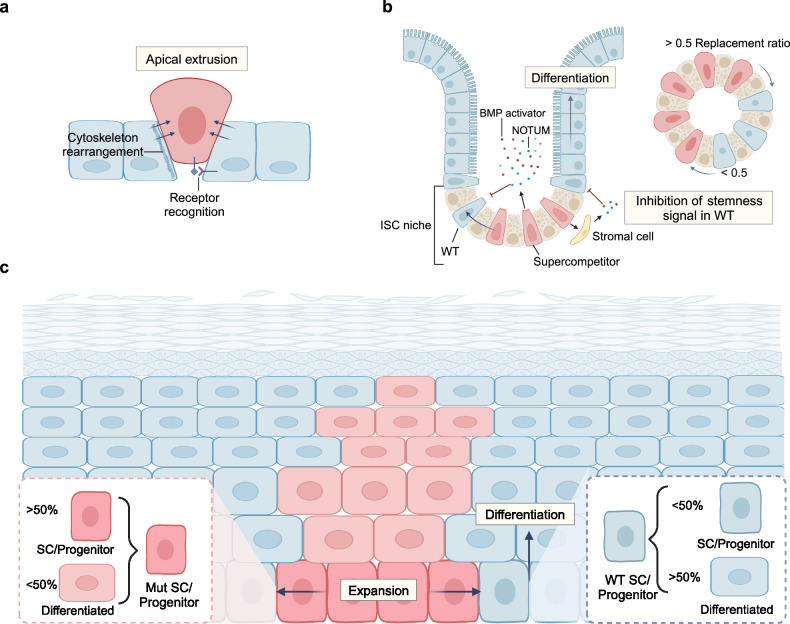
Cell competition across tissues. **a** Live cells can be extruded from simple intestinal epithelium by intercellular communications and cytoskeleton rearrangement. **b** Intestinal stem cells compete for dominance within the stem cell niche located at the bottom of the intestinal crypt. Mutant supercompetitors are more likely to maintain stemness, replace wild-type counterparts, occupy the ISC niche, and subsequently take over the entire crypt. The displaced wild-type cells, referred to as “losers,” differentiate, migrate upward along the crypt, and are ultimately shed at the top. The fate of stem cells can be regulated by secretory signals that come directly from supercompetitors and indirectly from stromal cells surrounding the crypts, stimulated by the supercompetitors. Stemness inhibitory signals, including BMP activators and NOTUM, differentially affect wide-type cells and supercompetitors by preventing wild-type cells from maintaining stemness, while having less effects on supercompetitors. **c** In stratified epithelium, the outcome of stem cell competition is also regulated by cell fate decisions. However, it is not limited to specific microstructure as the crypt, the winner clone has the potential to expanding to a large area. WT wild-type cells, BMP bone morphogenic protein, ISCs intestinal stem cells. Created with BioRender.com

The molecular mechanisms to elicit cell competition include mechanical force, cell-cell contact, and secretory signaling, and losers can be eliminated through various forms, including extrusion, apoptosis, differentiation, necroptosis, and entosis, which are quite different from one tissue to another.^267^ For instance, in mouse pancreas and intestinal epithelium, apical extrusion of living cells was employed to eliminate *Ras*-mutant cells, through intercellular communications and alterations in cytoskeleton^188,268^ (Fig. 5a). On the other hand, in self-renewing tissues, stem cell fate is a decisive factor for cell competition. Stem cells compete to occupy stem cell niche, and the winners have persistent self-renewal properties, while the differentiated cells would be removed from the stem cell niche. The structure of stem cell niches varies across tissue, which may be the cause for various clone sizes and structures in different tissues.^7^ In the intestinal glandular epithelium, the stem cell niche is located at the bottom of the crypt. Accordingly, competitions are confined to a single crypt and clones rarely expand to other crypts. Under normal conditions, intestinal stem cells (ISCs) stochastically differentiate and migrate upward along the crypt, shedding at the top. Otherwise, they maintain self-renewal and occupy the entire niche to form a monoclonal crypt, a phenomenon referred to as ‘crypt fixation’^269^ (Fig. 5b). Oncogenic mutations, such as *KRAS, APC*, and *PIK3CA*, have the potential to disrupt the neutral drift and tend to achieve crypt fixation.^270,271,272^ The scenario is different in stratified epithelium, where stem/progenitor cells are distributed throughout the entire basal layer without interference from microstructures. Therefore, fitter stem cells have the potential to expand across the entire structure theoretically, until they encounter cells with the same fitness and end the competition (Fig. 5c).

Recent studies indicated that oncogene-mutation supercompetitors have the ability to outcompete their wild-type counterparts by both rising their own fitness and decreasing their competitors fitness.^270^^,271^^,^^272^ *Apc*^*–/–*^ ISCs secrete notum palmitoleoyl-protein carboxylesterase, an antagonist of WNT signaling to inhibit wild-type ISC proliferation and to facilitate *Apc-*mutant clones towards crypt fixation, ultimately contributing to adenoma formation.^270,271^ Analogously, ISCs carrying *Pik3ca* or *Kras* mutation enhanced secretion of BMP ligand, mediating the differentiation of wild-type ISCs.^272^ Super-competition has also been observed in *Asxl1* CHIP, where mutant HSPCs generate mature offspring with elevated expression of pro-inflammatory genes.^273^ The inflammatory environment induced differentiation of wild-type cells, while the mutant HSPCs upregulated genes that suppress inflammation to protect themselves from differentiation.^273^

### Interactions with microenvironmental components

The microenvironment is composed of diverse immune cells, fibroblasts, and ECM,^274^ which have sophisticated interactions with transformed cells. On one hand, the healthy microenvironment plays a tumor-suppressive role and exerts the selective pressure to sculp clonal landscape. On the other hand, the transformed cells can remodel the surrounding niche to support their fitness, and accumulating work has identified early transformation of the microenvironment during tumorigenesis (Table 2). In this part, we aim to illustrate the interplays and co-evolutionary dynamics between mutant clones and their microenvironment during tumorigenesis.

#### Immune cells

The immune system possesses the capacity to suppress and shape tumors. Immune surveillance can be stimulated by mutation-induced neoantigens. Accordingly, immunogenic pressure selects for transformed cells that can evade immune recognition and killing, as well as those with the capability to sculp an immunosuppressive landscape.

**Fig. 6:**
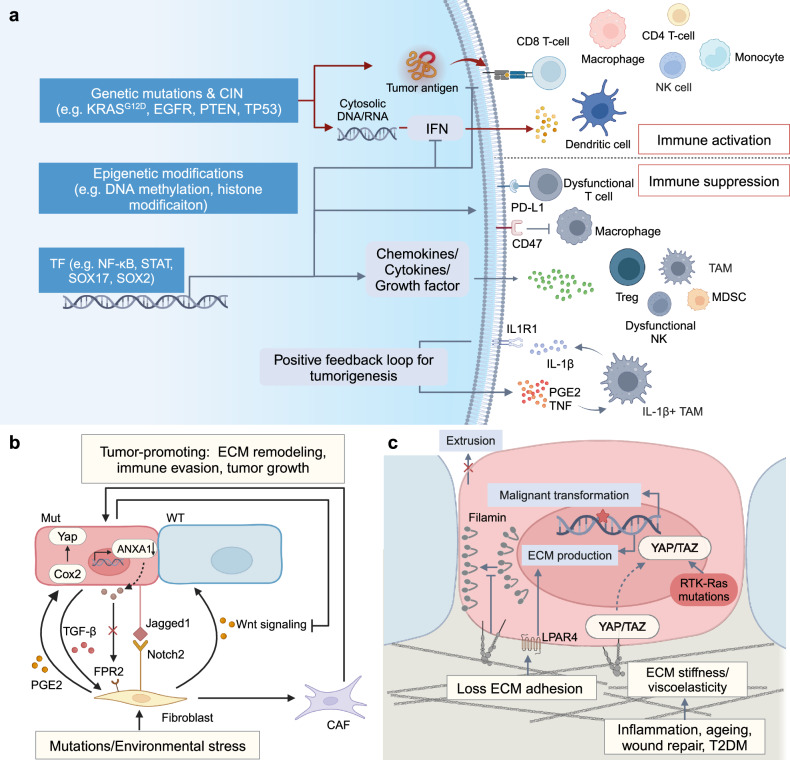
Interactions of transformed cell and microenvironmental components. **a** Abnormal genetic, epigenetic and transcriptional signals in transformed cells can paradoxically induce immune activation, while simultaneously developing strategies to achieve immune evasion. Their crosstalk is primarily mediated by direct cell-cell interaction signals and paracrine signals, such as chemokines, cytokines and growth factors and direct cell-cell interaction signals. As a positive feedback, tumor supportive immune cells, such as TAMs, which can produce IL-1β signals to further promote malignant evolution. **b** Transformed cells, along with environmental stress and genetic alterations, can activate fibroblasts through both secretory and contact signals, transforming them into CAFs with diverse tumor-promoting properties. In turn, fibroblasts secrete stemness signals to differentially regulate mutant and wild-type cells during cell competition. **c** Environmental signals can induce ECM remodeling, and a single transformed cell with ECM adhesion loss can also produce ECM to support its survival. In turn, abnormal mechanical signals in the ECM, including stiffness and viscoelasticity, under pathological conditions such as inflammation, aging, wound repair, and T2DM, predispose mutant cells to malignant progression through the activation of the YAP/TAZ pathway. The pro-tumorigenic effects can be aggravated by mutations in the RTK-Ras pathway. Additionally, a stiff ECM inhibits filamin from translocating from perinuclear areas to the interface of wild-type and mutant cells, further inhibiting the extrusion of mutant cells. TAMs tumor associated macrophages, MDSC myeloid-derived suppressor cell, cGAS cyclic GMP-AMP synthase, STING stimulator of interferon genes, IRF3 IFN regulatory factor 3, T2DM type 2 diabetes mellitus, Yap yes-associated protein, TAZ transcriptional co-activator with PDZ-binding motif, LPAR4 lysophosphatidic Acid Receptor 4. Created with BioRender.com

A convergent immune identity is present in almost all established tumors, including varying extents of suppression of cytotoxic T lymphocytes, natural killer cells, and dendritic cells, increases in regulatory T (Treg) cells and other suppressive cells, activation of pro-inflammatory cells, as well as transformation of myeloid cells into pro-oncogenic phenotypes^275–278^ (Fig. 6a). There is a continuum of immune evolution accompanying the transformation of cells from pre-cancerous stages (Table 3). For example, a stepwise process of CRC tumorigenesis was shown to be accompanied by a shift from pro-inflammatory to immune-suppressive macrophage populations, along with upregulation of ‘don’t eat me’ CD47-SIRPα signaling.^279^ Moreover, during the progression of preneoplasia to invasive LUAD, the immune system evolves with downregulation of immune-activation pathways, such as dendritic cell maturation and the acute phase reaction pathway, and upregulation of immunosuppressive pathways including T cell exhaustion signaling and poly adenosine diphosphate-ribose polymerase (PARP) signaling pathways.^280^ More importantly, the immune transformation may play a decisive role in the transition from precancerous lesions to tumors. Lung carcinoma in situ only progresses to cancer if immune evasion occurs while lesions with an active immune response and higher infiltration of CD8^+^ T cells would regress.^281^

As mentioned beforehand, many environmental factors change the immune landscape, stimulating chronic inflammation and increasing tumor susceptibility. In addition, the transformed cells can be a key driver of immune remodeling. Mechanically, tumor cells are able to regulate immune cell activation, chemotaxis, and polarization through paracrine secretion of cytokines, chemokines, and growth factors, or through direct cell-cell interaction signals, such as tumor antigens presented by major histocompatibility complex class l (MHC-I), programmed death ligand 1 (PD-L1), and CD47.^282^ In turn, a remodeled immune ecosystem supports further malignant progression. The crosstalk between transforming cells and the immune microenvironment is complicated and synergistically promotes the co-evolution. Caronni et al. found that transformed cells secreted high-level prostaglandin E2 and tumor necrosis factor (TNF) and thus promoted infiltration of IL-1β expressing tumor-associated macrophages (TAMs), which drove inflammatory reprogramming of neighboring transformed cells, resulting in a positive feedback loop to aggravate inflammation and tumor progression.^283^ Another case at this point is in *Hras*-mutant benign cutaneous papilloma. Upregulation of TGF-β pathway induced transcriptional reprogramming of cancer stem cells, resulting in upregulated expression of leptin receptors in cancer stem cells and angiogenesis.^284^ As a result, benign tumor cells enhanced sensing and responding to circulatory leptin levels, and activated downstream PI3K-AKT-mammalian target of rapamycin (mTOR) pathway, leading to malignant transformation.^284^

The immunomodulatory roles of transformed cells can be induced by genetic and epigenetic mutations and aberrate signaling. The driver mutations may serve as a major source of heterogeneity in the immune landscape of early tumors. Early transformation of host immunity in lung tumorigenesis was verified to be strongly associated with the type of driver mutations.^280^ Mutant *Kras* induced stronger immune activation compared with that of *EGFR* mutations from normal and premalignant to cancerous states, including CD8^+^ T cell infiltration, a low ratio of CD4^+^/CD8^+^ T cells and Treg/CD8^+^ T cells, and higher T cell clonality.^280^ Indeed, the immunomodulatory roles of the two classic tumor driver mutations have been widely explored. Cells harboring *Kras* mutation acquire capability to activate STAT3, secrete IL-6 and other proinflammatory cytokines. They also activate NLRP3 inflammasome and release chemokines, such as CCL5 and CXCL3, mediating tumor-promoting inflammation and immune modulation, and further promoting tumor progression.^285^ Similarly, *EGFR* mutations have been reported to promote Treg infiltration by upregulating CCL22 through activation of JUN amino-terminal kinase (JNK)/cJUN, and impede CD8^+^ T cell recruitment through downregulation of IRF1 and CXCL10 pathway.^286^ *Pten* deletion promoted PI3Kβ-mediated immune evasion through activation of the AKT and BMX-STAT3 pathways with reduced GM-CSF production, inactivation of dendritic cells, downregulation of antigen presentation pathways, and attenuation of IFNγ-mediated anti-tumor responses. In addition, mutations in *TP53*, another classical tumor suppressor gene, can not only maintain chronic inflammation by secreting IL-8 through the NF-κB pathway,^287^ but also inhibit innate immune response by disturbing the cytosolic DNA activated STING-TBK1-IRF3 pathway.^288^

In addition to genetic mutations, epigenetic and transcriptional factors are also involved in shaping the immune microenvironment. Repression of CXCL9 and CXCL10 expression, as well as impairment of CD8^+^ T cell infiltration in tumors, can be induced by mutations in isocitrate dehydrogenase and global hypermethylation.^289^ Meanwhile, oncogenic pathways, such as WNT-β-catenin, TGF-β, NF-κB and HIF, have the capability to alter the immune landscape by affecting the communication network between immune cells and cancer cells.^290^ A genome-wide CRISPR screening for genes modulating immune evasion from cytotoxic T lymphocytes in mouse cancer cells identified those involved in regulating IFN-response and TNF-induced cytotoxicity.^291^ Similarly, Martin et al. performed CRISPR screening in immunodeficient and normal mice, identifying multiple tumor suppressor genes that were positively selected by the adaptive immune system. These tumor suppressor genes are involved in various crucial cellular processes, such as chromatin interaction, antigen presentation, protein stability regulation, TGF-β signaling, and IFNα signaling.^292^ Although the evidence above is primarily based on research in established tumors, the effects of immune evasion are now being highlighted at the earliest stages of tumorigenesis. SOX17 deregulated IFNγ receptor expression and further lowered the expression of MHC-I and CXCL10, as well as CD8^+^ T cell infiltration. These changes played indispensable roles in the in vivo adaptation of genetically engineered naïve colon cancer organoids.^293^ In ESCC tumorigenesis, pathological overexpression of SOX2 activated endogenous retroviral elements and promoted double-stranded RNA formation, which should have activated immune surveillance.^294^ However, parallel upregulation of ADAR1 in turn attenuated the IFN signaling and contributed to immune escape.^294^ Interestingly, metabolic identities of tumor cells and immune components can also play a role in their interactions, forming competitive or dependent relationships with each other. On one hand, metabolites of tumor cells promote immunosuppressive effects,^295,296^ and in turn, phagocytosis of TAMs facilitates nutrient accumulation to meet energy requirement of tumor cells.^297^ On the other hand, there is nutritional competition between immune cells and tumor cells.^298^ mTORC1 signaling in TAMs plays a role in regulating the competition.^299^ Under normal protein diet conditions, the mTORC1 pathway is weakened in TAMs and thereby be enhanced in Myc-overexpressing tumor cells, resulting in a competitive advantage of tumor cells. Conversely, under low-protein diet conditions, activation of the GTPase-activating proteins GATOR in TAMs leads to TFEB/TFE3 nuclear translocation and mTORC1 activation in TAMs. As a consequence, TAMs gain an advantage over tumor cells in metabolic competition, exerting tumor-suppressive effects.^299^ Whether the mechanism is involved in early tumorigenesis warrants further exploration.

There are some arguments for the timing of immune activation and evasion. It is believed that there is an immune ignorance at the earliest cancerous stage where only a few transformed cells are present, and low levels of neoantigens they produced are deficient to activate immune clearance.^275,300^ The immune surveillance may not be a decisive factor for the initial clonal expansion.^41^ A mathematical model was developed to separate the fitness of driver mutations based on positive oncogenicity and negative immunogenicity. It revealed that *TP53* mutations in non-cancerous tissues were primarily selected for their pro-oncogenic proliferative advantage rather than negatively selected by immunogenicity.^301^ When progressing to advanced tumors, the pro-tumoral evolutionary force shifted into powering immune evasion. The shift could also explain the reason for different *TP53* hotspot mutations between cancer and normal tissues.^301^ The timing of switch from immune ignorance to activation and subsequent evasion need further exploration. High-resolution multiregional spatial and single-cell multi-omics sequencing are well suited to assess this issue. For instance, Cody et al. constructed a pseudo-temporal trajectory of colorectal tumorigenesis based on CIN and hypermutated pathways in their spatial multi-omic atlas, and mapped immune state changes along progression pseudotime, thereby facilitating prediction of immune exclusion.^302^

#### Fibroblasts

Fibroblasts constitute the primary stromal cellular components and serve major roles in ECM production, tissue structure maintenance, regulation of stem cells, interactions with immune cells, and participation in wound repair. Their role in regulating cell fate through paracrine orchestration can be hijacked by transformed cells to promote tumorigenesis^272,303^ (Fig. 6b). In the ISC niche, prostaglandin E2 secreted by a rare population of *PTGS2*-expressing fibroblasts can act on Sca-1^+^ ISCs and activate Cox2-Yap signaling for *Apc*^*Min/+*^ stem cell expansion and colon tumorigenesis.^303^ The stem cell niche signals produced by stromal cells also participate in the competition between oncogenic-mutant and wild-type cells. *Pik3ca* mutant ISCs showed an expansion advantage, partially by inhibiting stromal WNT signaling and creating a detrimental condition for the survival of wild-type ISCs.^272^

Alternatively, it is well-documented that cancer-associated fibroblasts (CAFs) are an important component in the TME. They can be activated by various stimuli in cancerous tissues, including TGF-β, inflammatory factors such as IL-1, IL-6, and TNF-α, physiological and genomic stress, ECM changes, and contact signals.^304–306^ In addition, CAFs promote tumor growth by remodeling the ECM, inducing immune evasion, and directly interacting with tumor cells.^304^ It has been confirmed that they emerge and contribute to the earliest stage of tumorigenesis (Table 3). The transformed cells are the main driver for CAF transformation.^307–309^ We identified a reciprocal mechanism between fibroblasts and epithelial cells, evolving synchronously in the multistep ESCC tumorigenesis.^307^ In the early stage of tumorigenesis, epithelial cells gradually downregulated *ANXA1* expression due to the suppression of transcription factor KLF4. Subsequently, the formyl peptide receptor type 2, an ANXA1 receptor on fibroblasts responsible for fibroblast homeostasis, was dysregulated and drove the transformation of CAFs. This process was accompanied by TGF-β secretion from transformed cells, further accelerating CAF transformation.^307^ Similarly, the epithelial-stromal interactions mediated by JAG1 on ductal carcinoma in situ cells and NOTCH2 on fibroblasts play a role in CAF transformation and mammary tumorigenesis.

Apart from transformed cells, other abnormal signals can prime pro-tumorigenic identity of fibroblasts before transformation. Mutations in fibroblasts, such as *BRCA1* and *NOTCH1* can also be regarded as the prerequisite of tumorigenesis. In addition, external stimuli, including aging, dense microenvironment and exposure to oncogenic insults can all promote transformation.^233,310–312^ Dermal fibroblasts under UV exposure induce suppression of NOTCH and its effector CSL, and promote the production of inflammatory cytokines, growth factors and matrix metalloproteinases, contributing to precancerous actinic keratosis lesions and CSCC formation.^313^

#### Extracellular matrix

The ECM is mainly composed of fibrous proteins and glycosaminoglycans, providing mechanical support, cellular anchoring, and storage for water and various bioactive molecules.^314^ Additionally, the ECM communicates with cells through local adhesions, converting chemical and mechanical signals into biological signals, regulating key cellular processes such as proliferation, apoptosis, fate decision, and migration. This process is known as mechanosensing and mechanotransduction.^315^ During tumorigenesis, the ECM experiences remodeling mainly driven by CAFs, tumor cells, and macrophages, leading to increased deposition, cross-linking and stiffness. As a result, the changes promote malignant progression by transducing abnormal biomechanical signals to transformed cells, as well as regulating immune recruitment and activation.^314,316^

The abnormal ECM has been profoundly investigated in established tumors, however, their earlier roles in regulating clone evolution before cancer formation and the accurate timing for oncogenic disorganization are unclear. Recently, Wu et al. reported the role of ECM remodeling in tumor initiation, where a solitary transformed cell at the very beginning of tumorigenesis met much more stress in a normal microenvironment than that in established tumors, including loss of cell-cell contact between tumor cells and pro-tumor ECM^317^ (Fig. 6c). The individual pancreatic cancer cell enhanced production of ECM and adapted to isolated stress by increasing expression of the stress-responsive gene lysophosphatidic acid receptor 4 (LPAR4) and promoted the production of fibronectin-rich ECM, which could compensate for the absence of stromal-derived factors and help tumor initiation.^318^ Furthermore, the ECM could also support neighboring cells without upregulated expression of LPAR4 through integrins α5β1 or αVβ3.^318^

Ahead of the transformed cell driven ECM remodeling, many pathological conditions, such as chronic inflammation, aging, and tissue injury, can increase stiffness of ECM and prime a tumor-vulnerable state.^319^ At the initial stage, stiffness influences the epithelial defense of oncogenic mutation. Filamin, an actin filament cross-linking protein located at the interface of wild-type and mutant cells, facilitates the extrusion of mutant cells under normal physiological conditions. However, when ECM is stiff, filamin relocates to the perinuclei and leads to the failure of epithelial defense against cancer and causes tumorigenesis.^320^ Furthermore, stiff signaling plays a role in cell fate regulation, and further regulates the susceptibility to oncogenic transformation.^321,322^ In the condition of *SmoM2* induced basal cell carcinoma, the back skin, which has a denser collagen I network compared with the skin of the ear, was not susceptible to the mutation-induced progenitor state reprogramming and tumor initiation.^321^ Chronic UV exposure and aging can decrease the expression of collagen, overcoming the natural resistance and increasing the risk of tumorigenesis.^321^ Orthogonally, oncogenic mutations render tumor-initiating cells to be more sensitive to signals of ECM stiffness. Even slight changes in ECM rigidity can trigger abnormal responses in cells harboring mutated oncogenes in the RTK-Ras pathway, such as human epidermal growth factor receptor 2 (*Her2)* and *Kras*.^322^ Stiffness and the mutations synergistically activated the YAP/transcriptional co-activator with PDZ-binding motif (TAZ) pathway, subsequently promoting the transformation of precancerous states.^322^ In addition to stiffness, viscoelasticity is another pro-tumorigenic mechanical property of ECM, which can be induced by advanced glycation end-products (AGEs) accumulation by type 2 diabetes mellitus. It is characterized by decreased interconnectivity of collagen matrix, shorter fiber length and greater heterogeneity, activating integrin-β1-tensin-1-YAP pathway and promoting cancer progression.^323^ Notably, YAP/TAZ serves as a molecular hub for mechanosensing and mechanotransduction, which is activated by mechanical signals transmitted by cytoskeletons, and is followed by the nucleus translocation and gene expression.^324^ Since abnormal YAP/TAZ pathway is strongly associated with various tumors,^324^ it is suggested that its oncogenic mechanotransductive signaling may be a general trait implicated in early malignant transformation. The interaction between mutations and mechanical signaling during tumorigenesis warrants further investigations.

### Tissue architecture restraint

**Fig. 7:**
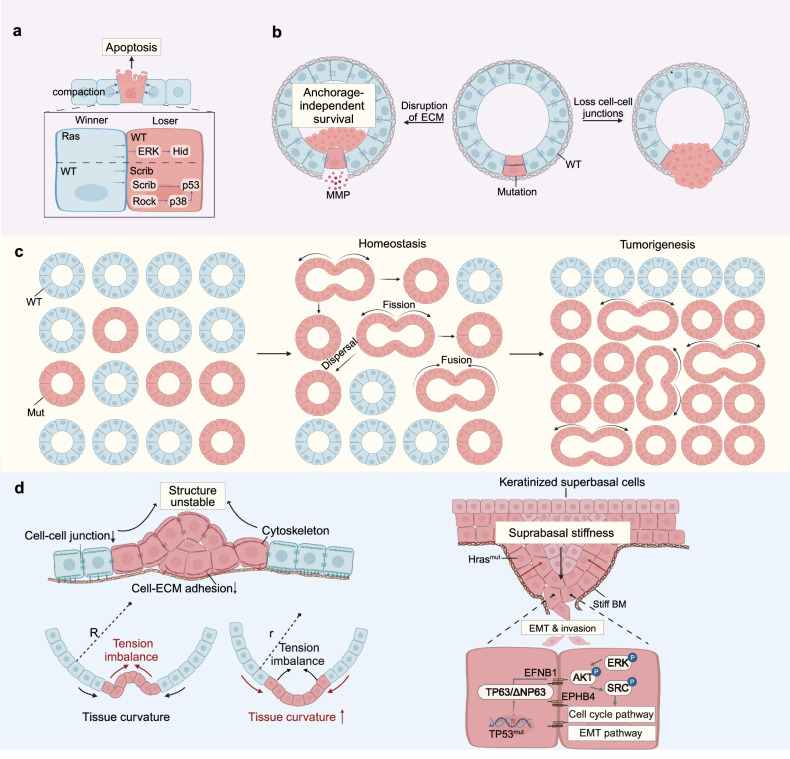
Tissue architecture restraint for clone expansion and alterations in tumorigenesis. **a** Cell density sensing mechanisms trigger apoptosis in cells sensitive to compaction, whether mutant or wild-type. **b** Mutations that disrupt the ECM and enable anchorage-independent survival allow cells to move to the lumen and expand. Additionally, the loss of cell-cell junctions can unleash the proliferative potential of mutations in situ instead of through translocation. **c** Mutant intestinal crypts are more likely to split rather than merge, increasing their number but still keeping overall balance through spreading and decreasing local crypt density. The *Kras* mutation speeds up this splitting, to a degree that cannot be counteracted by dispersal, leading to tumorigenesis. **d** When the homeostatic tissue architecture is disrupted, the mutant cells mediate a dysregulated tumor structure. This manifests as alterations in cell-cell junctions, cell-ECM adhesions and cytoskeleton rearrangement. The initial tissue curvature, as well as the stiffness of the basal membrane and suprabasal cells, all affect the nascent tumor morphogenesis. In addition, the EMT mediated by EFNB1-EPHB4 interactions in epithelial cells harboring *TP53* mutations occurs alongside early malignant morphogenesis. MMP metalloprotease, EMT epithelial-to-mesenchymal transition. Created with BioRender.com

Tissue structure is shaped by collective mechanical characteristics of individual cells, as well as their interactions with neighboring counterparts, stromal cells and the ECM. The maintenance of three-dimensional structural balance relies on stable number and arrangement of cells, which is also an important tumor-suppressive mechanism. Since there are tight interconnections and limited space in solid tissues, cell proliferation and elimination generate mechanical stress by the resistance of surroundings, thereby providing feedback to regulate cell behaviors.^325^ When over-proliferative cells cause density increase and compression, dense responsive signals are activated to suppress proliferation and eliminate redundant cells.^326^ Differential sensitivity to mechanical signals triggers cell competition.^327^ The mutations that endow cells with insensitivity to compression would be preserved (Fig. 7a). For example, when subjected to compression, *Scribble* mutant Madin-Darby canine kidney cells tended to undergo apoptosis due to p53 activation by ROCK and p38 pathways.^328^ On the contrary, *Ras*^*V12*^ mutant cells downregulated ERK in neighboring wild-type cells via competition, triggering apoptosis of wild-type cells.^329^

Furthermore, cell-cell junctions and cell-ECM adhesions are other important factors to arrest oncogenic growth and maintain homeostasis^326,330^(Fig. 7b). A well-organized acinar structure formed by a non-transformed human mammary epithelial cell line, MCF10A, remained quiescent in the presence of sporadic oncogenic mutations with proliferative potential until they expressed matrix metalloproteinases and disrupted cell-matrix adhesions. This disruption resulted in the translocation of mutant cells into the lumen, releasing more space for expansion.^330^ In addition, although detachment from the ECM alleviated the space limitation, the loss of the survival signal provided by the ECM would also lead to decreased fitness and apoptosis.^330,331^ Only cells that achieve anchorage-independent survival could continue to expand.^330^ Furthermore, the extrusion of mutant cells is also regulated by cell-cell junctions. Disruption of cell-cell junctions leaded to the transformation of the proliferative cells from lumen translocation to proliferation in situ.^330^

Some tissues have microstructures, which impose another barrier to the expansion of mutant clones.^37,332,333^ As mentioned earlier, the expansion of mutant stem cells is typically limited to a single intestinal crypt. Further expansion requires crypt fission, but it is a rare event for normal adult tissues, at approximately one fission every 27 years.^334^ Additionally, there are concurrent crypt fusions to maintain crypt density.^335^ Some mutations can break the balance and speed up crypt fission^333,336^ (Fig. 7c). This may account for discrepant elevations in the frequency of crypt fission without a concurrent rise in crypt fusion.^334^ Alternatively, dispersal of intestinal crypts occurs to counteract rising crypt density. However, the rate of crypt fission in *Kras* mutant crypts is too fast to be accommodated through dispersal, resulting in an increase in the local density of crypts, which increases the risk of polyps and tumor formation.^334^

Alongside overriding the structural restrictions of normal tissues, early tumor morphogenesis is shaped by cell proliferation, abnormal mechanics of transformed cells, and their microenvironment^337,338^ (Fig. 7d). *Ras* mutation induces MCF10A transformed cells to aggregate from two-dimensional (2D) to 3D structure through differential localization of E-cadherin at the top and bottom layers, reduction of adhesion to ECM, and redistribution of epithelial tension regulators. Neighbor structures of the lesion are also implicated. In tubular epithelia, whether lesion growth occurs outwards or inwards to the ductal lumen results from the balance between cellular tension of the lesion and the resistance of the tissue curvature.^338^ In stratified epithelium, the assembly of the basal membrane and the stiffness of superbasal layers also play a significant role in shaping tumors. Tumor budding is promoted by well-remodeled and soft basement membrane in *SmoM2* induced basal cell carcinomas (BCCs). By contrast, in *HRas*^*G12V*^ induced squamous cell carcinoma, stiffness from basal membrane and superbasal stratification promotes a folding architecture, which is more likely to develop an invasive tumor.^339^ However, molecular mechanisms underlying the gradual oncogenic tissue disorganization are not well understood. Based on spatial transcriptomic technology, our laboratory recently deciphered spatiotemporal expression patterns and identified key molecules driving the stepwise tissue destruction in esophageal tumorigenesis. Transformed cells interacted with each other through EFNB1-EPHB4 and triggered cell proliferation and EMT by SRC/ERK/AKT signaling, which were possibly instigated by ΔNP63 overexpression due to a *TP53* mutation.^259^

## Cancer risk prediction and intervention strategies

### Molecule-based cancer risk prediction

A better understanding of molecular and phenotypic determinants of malignant transformation facilitates cancer prevention, while the first step is to conduct risk assessment. Traditionally, it relies mainly on histopathological identification of precancerous lesions and combined demographic risk factors to identify individuals at high-risk of developing cancer, whereas predictive values are generally low. Only a small proportion of pathologically identified precancerous lesions progress to invasive tumors, inducing overdiagnosis and unnecessary interventions.^340^ In addition, as we have discussed above, some precancerous molecular alterations can emerge precedent or independent of morphological abnormalities. Therefore, molecular drivers identified in early tumorigenesis can be exploited to improve the efficacy of risk stratification, and further improve targeted surveillance and early interception.

Detection of germline mutations to evaluate inherited cancer susceptibility is widely explored, as exemplified by BRCA1 and BRCA2 pathogenic variations for breast and ovarian cancers.^341^ In the past few decades, large-scale case-control association studies across cancer types have facilitated the identification of cancer-risk loci and the development of polygenic risk scores for risk prediction.^342^ The combinations of polygenic risk scores and other known risk factors, including family history, lifestyle and reproduction, have been shown to accurately predict life-long risks of breast cancer.^343^ On the other hand, the pervasive existence of cancer driver mutations in normal tissues not only provides opportunities but also places higher demands for somatic molecule-based risk prediction, requiring accurately distinguishing between those as normal background and those as a consequential cancer signal during tumorigenesis. For example, in Barrett’s esophagus, *TP53* mutation and 17p LOH are relatively more specific predictors of progression to esophageal adenocarcinoma,^59,344^ with the *TP53* mutation even capable of predicting progression in samples with no dysplasia.^345^ Furthermore, a predictive panel of multiple driving mutations will have better performance than the *TP53* mutation alone.^346–349^ Another case of point is in CHIP, where it has been used in combination with hematologic and biochemical indicators to develop three independent risk prediction models for progressions to different myeloid neoplasms, including AML, myelodysplastic syndromes, and myeloproliferative neoplasms in a cohort of 454,340 UK Biobank participants, enabling early prediction of tumor occurrence in normal individuals.^350^ Given that CIN and CNAs are already present in specific precancerous diseases and accumulate throughout malignant progression, such as in Barrett’s esophagus, CNAs may also be a potential strategy for predicting risk of cancer.^351–354^ A genomic instability-based model was reported to distinguish patients with Barrett’s esophagus at high-risk of progression, among which 50% patients in the high-risk group were predicted 8 years before transformation of high-grade dysplasia or cancer.^351^ Furthermore, based on evolutionary measurement, genetic clonal diversity and clonal expansion are explored as predictive indicators of malignant evolution in colon, esophagus, and blood, potentially being a more universal method for various cancers.^355–359^ DNA methylation is also a promising type of risk prediction marker, demonstrating value in risk prediction for Barrett’s esophagus progression and gastric cancer formation.^360–363^ Liquid biopsy tests of circulating cell-free DNA fragments and/or their methylation patterns have gained widespread attention due to their non-invasiveness, low cost, and viable implementation. Tests for tumor DNA methylation have been validated in detecting multiple advanced cancers, whereas it appears to perform poorly in early-stage tumors.^364,365^ Advances in technology and more precises predictive panels are required to enhance this promising testing tool for use in premalignant stages.

Rapid development of high-throughput omics technologies in recent years has facilitated explorations of numerous biomarkers, and predictive panels based on transcriptomics, proteomics and metabolomics have been developed for specific tumors (Table 4). Based on serum metabolomics, lung adenocarcinoma and its preneoplasia can be distinguished from benign lesions by a metabolic panel.^173^ A gut microbiome-based panel has also shown efficacy in distinguishing CRC and adenoma from normal tissues, and further research is needed to verify its predictive role in disease progression.^202,203^ Based on multiplexed ion beam imaging by time of flight and tissue transcriptomics, Risom et al. mapped a spatial cellular landscape of ductal carcinoma in situ (DCIS) and delineated spatial and functional coordinated changes in stromal components from DCIS to invasive breast cancer, including myoepithelium, fibroblasts, and immune cells. Based on the features, they developed a risk prediction model for breast cancer invasion, which is largely dependent on myoepithelium and stroma. Intriguingly, disruption of myoepithelium indicates low risk of progression, which is contrary to the traditional belief that an intact myoepithelial barrier protects from tumor invasion, and the mechanism has not yet been detected.^366^ Altogether, multidimensional molecular features in the transition of tumors could be utilized to develop predictive assays. Nevertheless, most studies to date are based on small cohorts and sometimes lack validation cohorts, requiring further validations before being introduced into clinic.

**Table 4:** Molecular markers for cancer risk prediction

Class	Predictive markers	Usage	Refs
**Colon**			
Protein	Hemoglobin, fecal calprotectin	Diagnose high-risk adenoma and polyp	^506^
Genetic mutations	20 genes that differentiate conventional adenoma and non-hypermutated CRC	Distinguish adenoma and CRC	^347^
Bacteria	Fusobacterium nucleatum, hemoglobin	Detect advanced adenoma	^507^
Metabolites	Benzoic acid	Discriminate healthy controls and patients with adenoma	^508^
Metabolite	8 gut microbiome-associated serum metabolites altering in both CRC and adenoma	Discriminate CRC and adenoma from normal samples	^202^
Circulating immune cells	Relative FOXP3^+^ regulatory T cell counts	Predict risk of CRC	^509^
**Esophagus**			
Copy number variations	Gains of 1q, 8q, 9q, 12p, 20q, losses on 9p and 17p, aneuploidy and tetraploidy	Predict neoplastic progression in BE	^351–354^
Methylated DNA	*CDKN2A*, *RUNX3, HPP1, NELL1, TAC1, SST, AKAP12, CDH13* methylation	Predict neoplastic progression in BE	^362^
Methylated DNA	*UP10, UP35-1, CG6522, YPEL3, POU3F1* methylation	Discriminate NDBE from HGD and EAC	^363^
Methylated DNA	*TFAP2E, OTX1, OPLAH, CHAD, MARCH11, GALR1, HOXA9* methylation	Predict ESCC risk	^510^
**Blood**			
Genetic mutations	*DNMT3A, TET2* mutations, clinical characteristics (e.g. age, sex)	Predict risk of AML	^355^
Genetic mutations	CHIP gene mutations, variant allele frequency, clinical characteristics (e.g. age, sex)	Predict risk of AML, MDS and MPN	^350^
**Breast**			
TME	Coordinated spatial and functional changes in the TME of ductal carcinoma in situ	Predict risk of invasive progression from ductal carcinoma in situ	^366^
Genetic mutations	Loss-of-function variants in predisposition genes of breast cancer, polygenic risk score, clinical characteristics (e.g. family history)	Predict 2-year breast cancer risk	^511^
Metabolites	Histidine, N-acetyl glycoproteins, glycerol, ethanol	Predict breast cancer risk	^512^
Circulating immune cells	Relative CD8^+^ T cell counts	Predict breast cancer risk	^509^
**Lung**			
Protein	Pro-SFTPB, clinical characteristics (e.g. age, sex)	Predict lung cancer risk	^513^
Protein	CA125, CEA, CYFRA 21-1, pro-SFTPB, clinical characteristics (age, smoking)	Predict lone year lung cancer risk	^514^
Metabolite	Cystine, valine, asparagine, 3-chlorotyrosine, 12:0-carnitine, glutamate, phosphocholine	Distinguish invasive adenocarcinoma and its precursors with benign diseases	^173^
Gene expression	Genes in the airway field associated with premalignant lesions, such as *TOMM22* and *COX4I1*	Predict risk of lung premalignant lesion	^515^
Circulating immune cells	Relative counts of FOXP3^+^ regulatory T cells	Predict lung cancer risk	^509^
**Stomach**			
Serum	*H. pylori* IgG, pepsinogen I, pepsinogen I/II ratio, gastrin-17	Diagnose gastric precancerous lesions	^516,517^
Protein	APOA1BP, PGC, HPX, DDT, *H. pylori* infection, clinical characteristics (e.g. age, sex)	Predict gastric lesion progression	^518^
Methylated DNA	*TFAP2E, OTX1, OPLAH, CHAD, MARCH11, GALR1, HOXA9* methylation	Predict gastric cancer	^510^
**Cervix**			
Methylated DNA	*MAL* and miR-124-2 gene methylation	Detect cervical intraepithelial neoplasia	^519^
Methylated DNA	Number of hypervariable CpGs and hypermethylated CpGs associated with age	Predict risk of cervical neoplasia, detect pre-invasive neoplasia and cervical cancer	^520^
Serum	HPV DNA/mRNA, E6 oncoprotein, HPV genotyping, p16/Ki-67, clinical characteristics (e.g. age, cytology)	Predict risk of CIN2+	^521^
**Pancreas**			
Serum	IP-10, IL-6, PDGF, CA19-9	Discriminate pancreatic cancer from benign pancreatic disease	^522^

*CRC* colorectal cancer, *CHIP* clonal hematopoiesis of indeterminate potential, *AML* acute myeloid leukemia, *MDS* myelodysplastic syndromes, *MPN* myeloproliferative neoplasms, *BE* Barrett’s Esophagus, *ESCC* esophageal squamous cell carcinoma, *EAC* esophageal adenocarcinoma, *NDBE* non-dysplastic Barrett’s Esophagus, *HGD* high-grade dysplasia, *H. pylori Helicobacter pylori,* *CIN* Cervical Intraepithelial Neoplasia, *Pro-SFTPB* pro-surfactant protein B, *CEA* carcinoembryonic antigen, *CYFRA* cytokeratin fragment, *HPV* human papillomavirus, *PDGF* platelet-derived growth factor

### Intervention strategies

#### Chemoprevention

Chemoprevention refers to the use of synthetic or natural substances to reduce the risk of developing cancers (Table 5). The most popular chemoprevention strategy is endocrine therapy for breast cancer prevention. Indeed, endocrine therapies have been widely attempted for breast and prostate cancer prevention, by inhibiting binding of sex steroids and their receptors to block downstream gene regulation and tumor cell growth.^367^ Females with high-risk breast cancer are recommended to use selective estrogen receptor modulators, such as tamoxifen and raloxifene, or aromatase inhibitors, which inhibit aromatization of androgens and decrease the level of estrogens, but specific adverse events need considerations, including fracture, thrombosis, endometrial cancer, and cataract.^368,369^ In placebo-controlled randomized trials, tamoxifen can reduce the incidence of breast cancer by 31%, while raloxifene, aromatase inhibitors, exemestane and anastrozole, reduce it by 56% and 55%, respectively.^369^ They may also be effective in preventing DCIS.^369^ Similarly, 5α-reductase inhibitors, such as dutasteride and finasteride, have been attempted for prostate cancer prevention by inhibiting the synthesis of dihydrotestosterone, the most potent endogenous androgen.^370–372^ Although they have demonstrated an overall reduction in prostate cancer risk, the efficacy in high-grade prostatic tumor prevention requires further confirmation.^370–372^

**Table 5:** Potential agents for cancer prevention

Agent	Target	Mechanisms	Indication	Clinical evidence
Hormonotherapy				
Tamoxifen	Estrogen receptor inhibitor	Counteract estrogen effects and inhibit cell proliferation	Breast cancer	FDA approved^523^
Raloxifene	Estrogen receptor inhibitor	Counteract estrogen effects and inhibit cell proliferation	Breast cancer	FDA approved^523^
Anastrozole	Aromatase inhibitor	Inhibit the enzyme aromatase to reduce estrogen levels	Breast cancer	USPSTF recommend^368,369^
Exemestane	Aromatase inhibitor	Inhibit the enzyme aromatase to reduce estrogen levels	Breast cancer	USPSTF recommend^368,369^
Dutasteride	5α-reductase	Inhibit the conversion of testosterone to dihydrotestosterone	Prostate cancer	RCT^372^
Finasteride	5α-reductase	Inhibit the conversion of testosterone to dihydrotestosterone	Prostate cancer	RCT^371^
Anti-inflammation				
Aspirin	COX1/COX2	Inhibit prostaglandin synthesis, platelet activation, Wnt-β-catenin signaling, and inflammation	Colorectal cancer	USPSTF recommend^524^
			Barrett’s esophagus	RCT^525^
Sulindac	COX1/COX2		Colorectal polyps	RCT^526^
Celecoxib	COX2		Colorectal adenomas	RCT^527^
Small molecular inhibitor				
Ruxolitinib	JAK1/JAK2	Decrease STAT3 phosphorylation and induce cell apoptosis	Premalignant breast disease	Ongoing RCT (NCT02928978)
Erlotinib	EGFR	Induce growth inhibition, apoptosis and cell cycle arrest	Liver cancer	RCT (NCT02273362)
Metabolic agents				
Metformin	Mitochondrial complex I, MAPK and mTOR to modulate energy metabolism	Activate AMPK, which inhibits the mTOR pathway and reduces cyclin D1 expression and RB phosphorylation	Colorectal adenomas	RCT^383^
			Oral cancer	Ongoing RCT (NCT02581137, NCT05536037, NCT05237960)
			Lung cancer	Ongoing RCT (NCT04931017)
			Multiple myeloma	Ongoing RCT (NCT04850846)
Atorvastatin	HMG-CoA reductase	Disrupt the mevalonate pathway with downstream effects on membrane integrity, cell signaling, protein synthesis, and cell cycle progression	Colon cancer	Ongoing RCT (NCT04767984)
Immunotherapy				
HPV vaccine	HPV	Elicit both T-cell and antibody responses to HPV infected cells	Cervical intraepithelial neoplasia	FDA approved^523^
HBV vaccine	HBV	Inhibit the replication of HBV and promote its clearance	Primary liver cancer	RCT^528^
MUC1 vaccine	MUC1	Target aberrantly post-translationally modified antigen MUC1 in various cancer cells	Colorectal adenomas	RCT^407^
			Lung cancer	Ongoing RCT (NCT03300817)
			Ductal carcinoma in situ	Ongoing RCT (NCT06218303)
KRAS vaccine	KRAS	Target driver mutation	Pancreatic cancer	Ongoing RCT (NCT05013216)
EGFR vaccine	EGFR	Target driver mutation	Lung cancer	Ongoing RCT (NCT04298606)
HER-2/neu vaccine	HER-2/neu	Target driver mutation	Ductal carcinoma in situ	Clinical Trial^405^
Nivolumab	PD-1	Block the binding of PD-1 to PD-L1 and enhance the role of T cells in recognizing and killing tumor cells	Oral leukoplakia	RCT^416^
			Squamous dysplasia	Ongoing RCT (NCT03347838)
			Melanoma	Ongoing RCT (NCT04099251)
Pembrolizumab	PD-1		Oral Leukoplakia	Ongoing RCT (NCT03603223)
			Cervical intraepithelial neoplasia	Ongoing RCT (NCT04712851)
Calcipotriol+ 5-fluorouracil	TSLP	Induce TSLP expression and CD4^+^ T cell immune response	Skin cancer	RCT^411^
Micronutrient supplement				
Vitamin A	NA	Combine with retinol-binding protein to inhibit cell growth, induce differentiation, regulate cell-cycle-mediated stem cell plasticity	Skin cancer	RCDCIST^529^
Nicotinamide	NA	Boost cellular energy, enhance DNA repair and reduce level of immunosuppression	Nonmelanoma skin cancers, AK	RCT^445^
			Nonmelanoma skin cancer	RCT^530^
			Keratinocyte Carcinoma	Ongoing RCT (NCT05955924)
Vitamin D	NA	Bind to vitamin D receptors located in cell nuclei to inhibit proliferation and angiogenesis and induce differentiation and apoptosis	Colorectal adenomas	RCT^531^
			Hepatocellular carcinoma	Ongoing RCT (NCT02779465)
Calcium	NA	Bile acid-binding capacity, direct effect on calcium-sensing receptors on colonocytes	Colorectal adenomas	RCT^532^
Vitamin D+ calcium	NA	Reduce cell proliferation, induce differentiation and apoptosis, downregulate inflammatory mechanisms and regulate immune response	Breast cancer	RCT^448^
Folic acid	NA	Affect DNA replication, repair, and methylation through the one-carbon metabolic pathway	Colorectal adenomas	RCT^449^
			Cervical cancer	RCT (NCT00703196)
Long-chain omega-3 (PUFA)	Components of phospholipids that form cell membranes	Have potent anti-inflammatory effects, reduce cell proliferation and increase apoptosis	Colorectal adenomas; serrated polyps	RCT^441^
			DCIS; ADH	RCT (NCT00627276)
Selenium	NA	Depress carcinogen bioactivation, cell proliferation and cell cycling, increase apoptosis	Colorectal adenomas	RCT^533^
Squaric acid dibutylester	NA	Trigger innate immunity	Melanoma	Ongoing RCT (NCT04999631)
Berberine	NA	Change the structure of the microbiota, modulate the TME and block the activation of tumorigenesis-related pathways	Colorectal adenomas	RCT^442^

*FDA* Food and Drug Administration, *USPSTF* United States Preventive Services Task Force, *RCT* Randomized controlled trial, *COX* cyclooxygenase, *JAK* Janus kinase, *EGFR* epidermal growth factor receptor, *TSLP* thymic stromal lymphopoietin, *MAPK* mitogen-activated protein kinases, *mTOR* mechanistic target of rapamycin, *AMPK* AMP-activated protein kinase, *HMG-CoA* 3-hydroxy-3-methylglutaryl-coenzyme A, *HPV* human papillomavirus, *HBV* Hepatitis B virus, *DCIS* Ductal carcinoma in situ, *ADH* atypical ductal hyperplasia, *PUFA* polyunsaturated fatty acid, *AK* actinic keratoses

Given the important roles of inflammatory responses in tumorigenesis, anti-inflammatory regimens for cancer prevention are of great interest. Nonsteroidal anti-inflammatory drugs, especially aspirin, have shown preliminary efficacy in the prevention of various cancers, including those of the central nervous system, breast, esophagus, stomach, head and neck, liver, bile duct, colorectum, endometrium, lung, ovaries, prostate, and pancreas.^373,374^ Evidence for aspirin in preventing CRC is the most definitive. However, due to its severe adverse event of gastrointestinal bleeding, it is currently only recommended for Lynch syndrome and patients with removed familial adenomatous polyposis but is not routinely recommended for healthy individuals.^375–377^ Targeting key pro-carcinogenic inflammatory factors, such as IL-1, IL-6, and TNF-α, may enable more precise cancer prevention. In the cardiovascular CANTOS trial, the intervention arm using canakinumab, an IL-1β monoclonal antibody, significantly reduced lung cancer incidence.^378^ However, the costs and fatal adverse events of cytokine targeting therapy necessitate careful consideration for preventive applications.

Metformin is the first-line treatment for type 2 diabetes mellitus, primarily targeting molecules involved in energy metabolism, such as mitochondrial complex I, MAPK, and mTOR. It also plays a role in reducing insulin levels, enhancing insulin sensitivity and exerting effects on immune cells.^379,380^ In cell competition models, metformin reverses insulin resistance or enhances aerobic glycolysis, eliminating the competitive advantage of mutant cells, suggesting its potential inhibitory effect on tumor initiation.^184,381^ Since a preliminary retrospective case-control study in Scotland was reported in 2005, the preventive use of metformin for tumors has been supported by several observational studies.^382^ A randomized controlled trial in Japan confirmed the protective role of metformin from adenoma and polyp recurrence^383^; however, there is a lack of further evidence from intervention trials for the reduced risk of various cancers with metformin use.^384^ It is hypothesized that personalized regimens of metformin may be necessary, in order to particularly target tumors that are dependent on oxidative phosphorylation, as metformin primarily targets mitochondrial respiration. Additionally, due to metabolic reprogramming of tumor cells after metformin treatment, combination therapy targeting metabolic pathways on which tumor cells depend may enhance metformin efficacy.^380^ Another focus of metabolic regulation is statins, a class of drugs used to treat lipid disorders. As inhibitors of 3-hydroxy-3-methylglutaryl coenzyme-A (HMG-CoA) reductase (HMGCR), statins are associated with reduced overall cancer risk, and liver, prostate, lymphoma, and CRC risks.^385–393^ Statins inhibit de novo cholesterol synthesis via the mevalonate pathway and promote the removal of plasma low-density lipoprotein cholesterol by acting on low-density lipoprotein receptors. Mechanistically, statins exert anti-tumor effects by promoting cell death, regulating angiogenesis, and modulating immunity.^394^ In early stages of colorectal tumorigenesis, they can also modulate gut microbiota.^395^ Treatment with statins increases microbial tryptophan availability in the gut, promoting the growth of *Lactobacillus reuteri*, which converts tryptophan into indole-3-lactic acid and regulates Th17 cells to inhibit tumor formation. More evidence is required to support their clinical use.

A deeper understanding of molecular events in early tumorigenesis offers insights into novel interventional targets. Accurately identifying the cell state at the root or pivotal transitional point along the pathway of malignant transformation is essential for searching for potential targets within these cells. For instance, compared with wild-type luminal epithelium, the mammary epithelium in individuals carrying BRCA2 mutations exhibits an increased number of ERBB3^lo^ luminal epithelial cells, which potentially serve as the cells of origin for both ER^+^ and ER^-^ breast cancers.^396^ mTORC1 signaling is significantly upregulated in these cells. Short-term treatment with a mTORC1 inhibitor substantially curtailed tumorigenesis in a preclinical model, thus uncovering a potential strategy for *BRCA2* breast cancer prevention.^396^ In tobacco-induced LUAD, considering that the identified progenitor cell subset harbors *KRAS* mutation, it is logical to hypothesize that *KRAS* mutation inhibitor can play a role in intercepting the earliest tumorigenesis.^253^ Similarly, as *TP53* mutations are widespread in normal tissues and are identified as the early key driver events in various tumor initiation, there are many explorations for *TP53* targeting strategies. Methods such as blocking murine double minute 2 (MDM2), as well as restoring dysfunctional p53 are being attempted.^397^ It is expected to identify more promising targets and accordingly develop robust agents in the future.

#### Immunoprevention

Immunoprevention involves modulating the host immune system to elicit early anti-tumor immune responses, eliminating tumor cells at precancerous stages. The most classic example of immunoprevention is vaccination against carcinogenic pathogens, such as human papillomavirus for preventing and treating cervical intraepithelial neoplasia,^398,399^ and HBV for preventing hepatocellular carcinoma.^400–403^ In addition to viral-based vaccines, vaccines targeting tumor antigens have been attached great attention in recent years. The targets can be tumor-associated antigens abnormally overexpressed in tumor cells compared to normal tissues, such as carcinoembryonic antigen (CEA) and HER2, or aberrantly post-translationally modified antigens, such as Mucin 1 (MUC1).^404^ Among them, *HER2*-based vaccines have preliminarily shown success in interception of DCIS in clinical trials.^405,406^ In addition, since aberrant hypoglycosylation of MUC1 occurs in precancerous lesions of multiple epithelial cancers, there are many attempts of vaccines targeting MUC1 in clinical trials for various cancers.^404^ However, it was recently reported that there were limited effects of MUC1 vaccine in preventing colonic adenomas in a randomized controlled trial. Individuals with advanced adenoma received MUC1 peptide vaccine within 1 year after adenoma removal. Despite eliciting significant antigen-specific immune responses, adenoma recurrence did not significantly decrease.^407^ Therefore, further study is still required to improve vaccine efficacy for preventive usage. Another strategy is to target tumor-specific driver mutations that have already occurred in precancerous stages, such as *KRAS* and *EGFR* mutations for lung cancer prevention.^408–410^ These tumor-specific antigens are likely to be more immunogenic with better responses, but clinical trials are required to verify their efficacy and safety. Clinical trials on the mutant *KRAS*-targeted long peptide vaccine for high-risk pancreatic cancer recipients and *EGFR*-targeted vaccine for high-risk lung cancer recipients are currently underway (NCT05013216, NCT04298606).

As described above, immunosuppressive microenvironment has emerged at an early stage of tumorigenesis; accordingly, immunomodulatory strategies are being attempted for tumor prevention. For precancerous actinic keratosis of the skin, a combination of calcipotriol and 5-fluorouracil was adopted in a randomized controlled trial, which can induce squamous cell expression of thymic stromal lymphopoietin, thereby mobilizing anti-tumor immunity. Compared to using 5-fluorouracil alone, the combination showed significant lesion reduction, accompanied by upregulation of thymic stromal lymphopoietin, HLA-II, natural killer cell group 2D ligand expression, as well as CD4 T cell infiltration.^411^ Long-term follow-up indicated that the effects of immunomodulation persisted three years later, with a decrease in the incidence of CSCC.^412^ PD-L1 and PD-1 upregulation has been observed in precancerous lesions of the oral cavity^413,414^ and lung tumors,^415^ suggesting that PD-1 monoclonal antibodies are an ideal early immunoprevention strategy. Currently, relevant clinical trials are underway (NCT03347838, NCT03603223). In a preliminary trial to evaluate the safety and clinical response of anti-PD-1 therapy among patients with high-risk proliferative verrucous leukoplakia, 12 patients (36%) (95% CI, 20.4%-54.8%) had a > 80% decrease in size and degree of dysplasia after receiving nivolumab, suggesting potential clinical activity for nivolumab in high-risk proliferative verrucous leukoplakia.^416^

#### Lifestyle and dietary interventions

Lifestyle and dietary interventions are low-cost, low-risk, and accessible preventive strategies. There are various advocated healthy lifestyles against cancers, including avoiding and ceasing exposure to carcinogens such as tobacco, alcohol, and UV, as well as adopting healthy diets and engaging in regular exercise.^417–420^ In terms of dietary interventions, multiple healthy dietary patterns, such as the Mediterranean diet, vegan diets, and various healthy diet guideline indices have been proposed.^417^ Their core tenets are avoiding carcinogenic dietary components, controlling total calories, and increasing proportions of beneficial constituents.^421^ However, most evidence is based on epidemiological associations from population studies, and many confounding factors cannot be excluded.^421^ Exploring the molecular mechanisms behind specific nutrients in healthy diets for cancer prevention can not only strengthen the evidence supporting existing dietary interventions, but also yield insights for developing novel and scientifically grounded strategies. Low calorie intake and various fasting regimens are confirmed to inhibit nutrient sensing pathways and activate nutrient scarcity sensors to regulate cellular stress responses, modulating tumor cell activity and anti-tumor immune response.^422^ Another popular regimen is ketogenic diet, which means to intake low carbohydrates, high fat, and moderate protein to enhance ketone metabolism. Some clinical trials have confirmed its therapeutic effects in patients with breast cancer undergoing chemotherapy or radiotherapy.^423–425^ Although there is a lack of clinical evidence to support the preventive usage of ketogenic diet, its benefits have been shown in preclinical models. Ketogenic diet induced-β-hydroxybutyrate could bind the Hcar2 receptor on intestinal stem cells and activate tumor suppressive TF Hopx to inhibit cell proliferation and exert anti-cancer effects.^426^ Furthermore, since oral supplement of β-hydroxybutyrate alone could achieve an anti-tumor effect, it may be served as an alternative regimen for the ketogenic diet, possibly addressing the issue of low compliance with strategies that change the overall dietary pattern.^426^ Specific diets may also act as prebiotics or probiotics.^427^ The most popular one is high-fiber diets, which are associated with a lower risk of multiple cancers.^428–433^ Dietary fiber can be fermented into short-chain fatty acids by microbes to regulate microbe composition and diversity, protect intestinal mucus barrier, and prevent bacterial translocation, thereby modulating systemic metabolism, immunity, and inflammation.^434–437^ A small randomized cross-over trial confirms that supplementing fermentable fiber inulin and inulin-propionate ester, which is aimed at delivering short-chain fatty acids to the colon, can modulate gut microbes, metabolism, and inflammation, thereby improving insulin resistance.^438^ Recently, the BE GONE trial confirmed similar findings through the supplementation of beans, a fiber-rich food. Soybeans can act as prebiotics, regulating gut microbes, inflammation, and metabolism, improving biomarkers of metabolic obesity and colon cancer.^439^ However, direct evidence from fiber intervention trials for cancer prevention is still lacking. Another study held an opposite conclusion. It found that high-dose soluble fiber could dysregulate gut microbiota and metabolites, leading to enrichment of potentially pathogenic bacteria and depletion of probiotics, and prompt colorectal tumorigenesis.^440^ Specific effects of high-fiber diets are still warranted to be further explored.

Apart from adjusting dietary structure and macronutrient intake, direct supplementation of specific anti-tumor nutrients and metabolites is a more implementable strategy. Given the epigenetically tumor-suppressive effects of α-ketoglutarate demonstrated in mouse models, dietary supplementation of its precursor molecule glutamine may be a potential preventive strategy.^177^ Other dietary supplements, including marine omega-3 fatty acids sourced from fish and seafood,^441^ as well as the plant-derived natural alkaloid berberine,^442^ have demonstrated preliminary efficacy in preventing CRCs. Further large-scale clinical trials and long-term follow-up are required. On the other hand, various vitamins have been proposed for tumor prevention, and some of them have illustrated promising applications. Nicotinamide, which belongs to vitamin B3 family, plays a role in inhibiting oxidation and DNA damage.^443,444^ A Phase III clinical trial showed that it can effectively reduce the risk of non-melanoma skin cancers and actinic keratoses in high-risk populations.^445^ However, it failed to show a preventive effect in immunocompromised individuals following organ transplantation, possibly due to DNA damage resulting from the use of immunosuppressive drugs.^446^ In addition, low-dose acitretin, a vitamin A derivative, has been demonstrated to have a preliminary preventive effect on skin cancer in organ-transplanted recipients. Renal transplanted patients with actinic keratosis received acitretin therapy (20 mg/d) for 1 year and there was an improvement of actinic keratosis in all patients.^447^ Mechanistically, acitretin exerts an anti-tumor effect by increasing the number of epidermal Langerhans’ cells and enhancing skin immune monitoring.^447^ Other examples of effective preventive strategies include vitamin D for DCIS and high-dose folic acid for recurrent colorectal adenoma.^448,449^ However, these clinical trials are limited by their small scale and short-term follow-up. Apart from the examples mentioned above, there is a notable scarcity of successful cases in interventions using other vitamins and micronutrients.^450,451^ This underscores the need for more high-quality retrospective and prospective studies to evaluate the potential impacts of micronutrients. Such studies should be conducted in conjunction with preclinical research that demonstrates molecular mechanisms, thereby facilitating the identification of compounds suitable for future dietary interventions.

## Conclusion and future perspectives

Driven by genetic and epigenetic alterations along with environmental signaling, transformed cells not only acquire cell-intrinsic proliferative advantages, but also actively remodel their environment to support their aberrant behaviors during early tumorigenesis. Encouragingly, apart from mutagenesis, many determinants of tumorigenesis are reversible, and understanding the molecular mechanisms underlying early malignant evolution provides significant translational opportunities. Cancer prevention aims to identify high-risk individuals and implement early interventions with high efficacy, low adverse events, and the potential to cure. Since many targetable aberrative pathways in advanced tumors have also been found in the earliest stages, including those affecting the cell cycle, anti-apoptosis, metabolic remodeling and immune evasion, classic anti-tumor agents might be repurposed for earlier interventions. However, extensive clinical trials to verify their efficacy and safety are warranted, and the balance of expenses and benefits should be considered.

Tumors originate from individual cells, presenting significant challenges in capturing these rare cell subsets. Advances in next-generation sequencing, single-cell, and spatial omics have revolutionized the study paradigm of tumorigenesis. At an extremely high resolution, precursor clones of various tumors have been identified, and co-evolutionary dynamics of the transformed cells and their microenvironment are being depicted. Furthermore, integrative analyses of paired omics modalities, such as genome, epigenome, and transcriptome, have been preliminarily applied to map the early tumorigenesis events,^117,135,302,452^ offering insights into the ordering and interplays among multiple evolutionary drivers, as well as their roles in regulating cellular phenotype. As multiplexing spatial and single-cell multi-omics technologies continue to enhance their throughput, resolution, and accuracy, coupled with innovations in bioinformatics tools to analyze unprecedented high-dimensional data,^453^ it is anticipated that multi-omics approaches can be leveraged to achieve a more comprehensive understanding of the complex biological processes of tumorigenesis.

To date, many studies primarily infer evolutionary trajectories computationally from multisampling of cancer specimens. However, this approach is limited to capturing only the dominant malignant clones and their major driver events. There is a loss of information regarding dynamic precancerous clonal competition and selection, since other precancerous clones may have been swept out in advanced tumors. Therefore, the importance of employing multiple sampling strategies to cover various stages of the malignant continuum is being increasingly recognized. Specifically, acquiring both cancerous and non-cancerous clones with shared ancestors simultaneously can optimize phylogenetic analysis results, depicting both the malignant evolutionary dynamics and the fate of remaining non-cancerous clones with partially shared mutations. This approach highlights the additional changes necessary for evolution into a malignant phenotype and their sequence among various driver events.^454^ Given that there are some premalignant stages that do not progress to invasive tumors, it is emphasized that rational cohort design in longitudinal studies to distinguish premalignant lesions from regression to progression can indicate key mechanisms that ultimately drive tumorigenesis. Yet, significant challenges remain in ensuring patient compliance and completely removing precancerous lesions during initial sampling, which usually aims at prevention and may interrupt natural disease progression.^455^ Alternatively, inducing autochthonous tumors in animal models or organoids offers an alternative way to study the early evolutionary processes. By prospectively introducing driver events informed by prior knowledge, and integrating lineage tracing with in vivo imaging techniques, real-time clone dynamics and their temporal evolutionary trajectory are visible, further facilitating the study of biological functions of specific perturbations in early tumorigenesis. At this point, Yao et al. recently reported their protein level reporter system, which is capable of tracing mutant p53 protein accumulation, a cancer-specific event as well as a potential mark for early transformed cells.^456^ The system sensitively identified rare precancerous cells in noncancerous tissues, and further facilitated characterization of cellular phenotypes underlying transformation, as well as the identification of potential interventional targets.^456^ In the future, a deeper understanding of the ordering and interactions of the driver molecular events, and their dynamic evolution under varying local and systemic environmental pressures and during specific tumorigenic phases, will help us gain more insights into tumor prevention, diagnosis, and early intervention.
